# Conserved non-AUG uORFs revealed by a novel regression analysis of ribosome profiling data

**DOI:** 10.1101/gr.221507.117

**Published:** 2018-02

**Authors:** Pieter Spealman, Armaghan W. Naik, Gemma E. May, Scott Kuersten, Lindsay Freeberg, Robert F. Murphy, Joel McManus

**Affiliations:** 1 Department of Biological Sciences, Carnegie Mellon University, Pittsburgh, Pennsylvania 15213, USA;; 2 Computational Biology Department, Carnegie Mellon University, Pittsburgh, Pennsylvania 15213, USA;; 3 Illumina, Incorporated, Madison, Wisconsin 53719, USA

## Abstract

Upstream open reading frames (uORFs), located in transcript leaders (5′ UTRs), are potent *cis*-acting regulators of translation and mRNA turnover. Recent genome-wide ribosome profiling studies suggest that thousands of uORFs initiate with non-AUG start codons. Although intriguing, these non-AUG uORF predictions have been made without statistical control or validation; thus, the importance of these elements remains to be demonstrated. To address this, we took a comparative genomics approach to study AUG and non-AUG uORFs. We mapped transcription leaders in multiple *Saccharomyces* yeast species and applied a novel machine learning algorithm (uORF-seqr) to ribosome profiling data to identify statistically significant uORFs. We found that AUG and non-AUG uORFs are both frequently found in *Saccharomyces* yeasts. Although most non-AUG uORFs are found in only one species, hundreds have either conserved sequence or position within *Saccharomyces*. uORFs initiating with UUG are particularly common and are shared between species at rates similar to that of AUG uORFs. However, non-AUG uORFs are translated less efficiently than AUG-uORFs and are less subject to removal via alternative transcription initiation under normal growth conditions. These results suggest that a subset of non-AUG uORFs may play important roles in regulating gene expression.

Gene expression is regulated at multiple levels, including transcription, translation, and turnover of mRNA and proteins. Each layer of regulation is modulated by *cis*-acting sequence elements encoded in DNA, mRNA, and protein, respectively. Advances in high-throughput approaches (e.g., ChIP-seq, ATAC-seq) have allowed comprehensive annotation of *cis-*acting DNA sequences that may influence transcription. Similarly, identifying *cis*-acting RNA elements that influence translation has been accelerated through the application of Ribosome profiling (Ribo-seq) (Ingolia et al. 2014), which measures ribosome occupancy throughout the transcriptome at near nucleotide precision. Common to these approaches is the need for careful statistical control and validation of proposed regulatory elements.

Ribo-seq has enabled the widespread identification of upstream Open Reading Frames (uORFs). Located within transcript leaders of protein coding genes, uORFs are *cis*-regulatory elements that affect translation of the downstream main ORF (Somers et al. 2013; Wethmar 2014; Hinnebusch et al. 2016). uORF regulatory effects can be conceptualized through the scanning model of translation initiation, in which the preinitiation complex binds the 5′ end of transcript leaders and scans downstream in search of start codons (Hinnebusch and Lorsch 2012). If initiation occurs at a uORF, there are two possible outcomes: The uORF can act as a repressor of translation of the main ORF, potentially triggering mRNA turnover through nonsense-mediated decay; alternatively, uORFs can act as stress-specific enhancers by promoting downstream reinitiation at main ORFs. For example, *GCN4* transcripts in *Saccharomyces cerevisiae* host four AUG uORFs, numbered sequentially from the 5′ end. During growth in rich media, ribosomes translate uORF1 and reinitiate at the repressive uORFs 2–4. Under starvation conditions, ribosomes bypass uORFs 2–4 and reinitiate at the main ORF (Hinnebusch 2005). A similar paradigm is used to regulate stress-responsive translation of the mammalian transcription factor ATF4, and many mRNAs that are well-translated under stress rely on reinitiation downstream from uORF translation (Andreev et al. 2015). uORF-translation in the cauliflower mosaic virus has also been shown to promote shunting past a very large structured region of viral RNA (Ryabova and Hohn 2000). Other work has identified uORFs with additional functions, including constitutive enhancement, and stress-dependent repression of protein production (Waern and Snyder 2013). Consequently, uORFs are versatile, context- and condition-specific regulators of mRNA translation and turnover.

Several studies have evaluated uORF evolution by analyzing conservation of the upstream AUGs that most often function as uORF translation initiation sites (TIS) (Fang et al. 2000; Cvijović et al. 2007; Hood et al. 2009; Selpi et al. 2009; Wethmar 2014; Chew et al. 2016; Johnstone et al. 2016). AUG uORFs typically act as repressors, and mutations that introduce novel AUG uORFs are likely to be detrimental. Indeed, the canonical start codon sequence “AUG” is significantly depleted within transcript leaders (Saito and Tomita 1999; Rogozin et al. 2001), suggesting that purifying selection may eliminate many AUG uORFs. Consistent with this reasoning, AUG uORFs not removed by purifying selection are enriched in alternative transcript leader regions (Resch et al. 2009; Pelechano et al. 2013; Waern and Snyder 2013), implying that they are regulated by alternative transcription initiation and splicing. Interestingly, AUG uORFs are also enriched in genes that regulate expression networks (Davuluri et al. 2000), and many uORFs that function during vertebrate development are highly conserved (Chew et al. 2016; Johnstone et al. 2016). Together, these studies are consistent with the view that uORFs can be important translational repressors.

Intriguingly, there have been hints that uORFs may be more common than initially believed. Translation can initiate (most-likely using tRNA_i_^Met^) at near cognate codons (NCCs)—sequences that differ from the canonical AUG start codon by one nucleotide (Zitomer et al. 1984; Peabody 1989; Ivanov et al. 2008, 2011; Kolitz et al. 2009; Starck et al. 2012; Kochetov et al. 2013; Gao et al. 2014). Thus, many NCC uORFs may be lying hidden in eukaryotic genomes. Analyses of Ribo-seq data have since resulted in thousands of NCC uORF predictions (Ingolia et al. 2009, 2011; Brar et al. 2011; Lee et al. 2012; Andreev et al. 2015). However, NCC uORF predictions have been made primarily through heuristic analysis of ribosome profiling data without statistical control, leaving questions as to the functional significance of these predicted uORFs. Here, we address these questions using comparative genomics. We combined multiple high-throughput approaches and a novel machine learning algorithm to identify and compare thousands of statistically significant AUG and NCC uORFs in multiple *Saccharomyces* species.

## Results

### Mapping *Saccharomyces sensu stricto* transcription start and end sites

**Figure 1.**
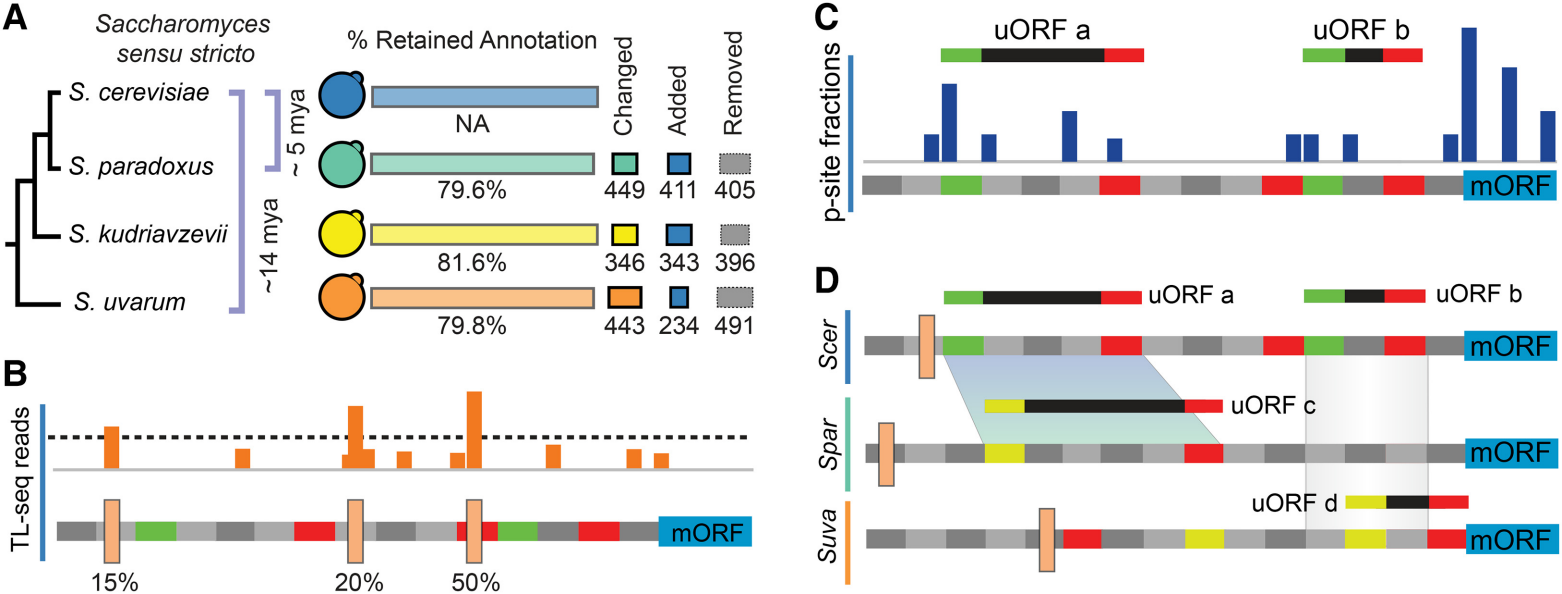
Overview. (*A*) The genomes of nonmodel species were reannotated to include ncRNA and correct previous errors, resulting in changes to ∼20% of genes on average (Methods). (*B*) Transcript leaders and polyadenylation sites were annotated for four *Saccharomyces sensu stricto* species using TL-seq (Arribere and Gilbert 2013) and pA-seq (Yoon and Brem 2010). TL-seq and pA-seq peaks were trend filtered for FDR control (β = 0.001) and evaluated for their usage frequency. (*C*) Ribosome profiling data from *S. cerevisiae*, *S. paradoxus*, and *S. uvarum* were analyzed to quantify the locations of ribosome P-site occupancy (dark blue peaks). These results were used to identify candidate uORFs. (*D*) Candidate uORFs were evaluated and scored using the uORF-seqr machine learning algorithm. uORFs with significant scores were retained for further analysis. The transcript leaders of gene homologs were aligned to identify uORF homologs (marked by shaded maps). These were identified by both local sequence alignment (uORFs a and c) and by positional overlap (uORFs b and d).

We chose *Saccharomyces* yeast to study NCC and AUG uORFs because of their simple gene structure (rare alternative splicing) and high-quality genome sequences (Scannell et al. 2011). However, except for *S. cerevisiae*, gene annotations for these species often lack start codons, stop codons, or both, and transcript boundaries have not been annotated. We used a pairwise, synteny-sensitive, alignment method to reannotate the genomes of *S. paradoxus*, *S. kudriavzevii*, and *S. uvarum* (Fig. 1; Supplemental File 1A–D). We next mapped transcript leader sequences (TLSs) and 3′ UTRs genome-wide for each of the four species using high-throughput sequencing approaches, TL-seq (Arribere and Gilbert 2013) and pA-seq (Yoon and Brem 2010). We identified significant (false discovery rate [FDR] < 0.001) (Methods) transcription start sites (TSSs) and polyadenylation sites (pAs) in all four species (Supplemental File 1A–D). These improved gene and transcript annotations should be a valuable resource to the research community, and they enabled us to study transcript leader and uORF evolution.

### Evolutionary comparisons of yeast transcript leaders

Although it is well known that *S. cerevisiae* uses alternative TSSs and pAs to generate diverse transcript isoforms, it is unknown how conserved this is across the *sensu stricto* clade. Because transcription initiates in short regions of the genome, we clustered our observed sites in 25-nt windows. Genes with more than one cluster of TSS or pA sites can be defined to have alternative TSS or pA regulation, respectively. By this measure, 48% of genes maintained alternative TSS regulation across all four species, and 65% maintained alternative pA regulation. This suggests that alternative transcription initiation and polyadenylation are often conserved in these species.

**Figure 2.**
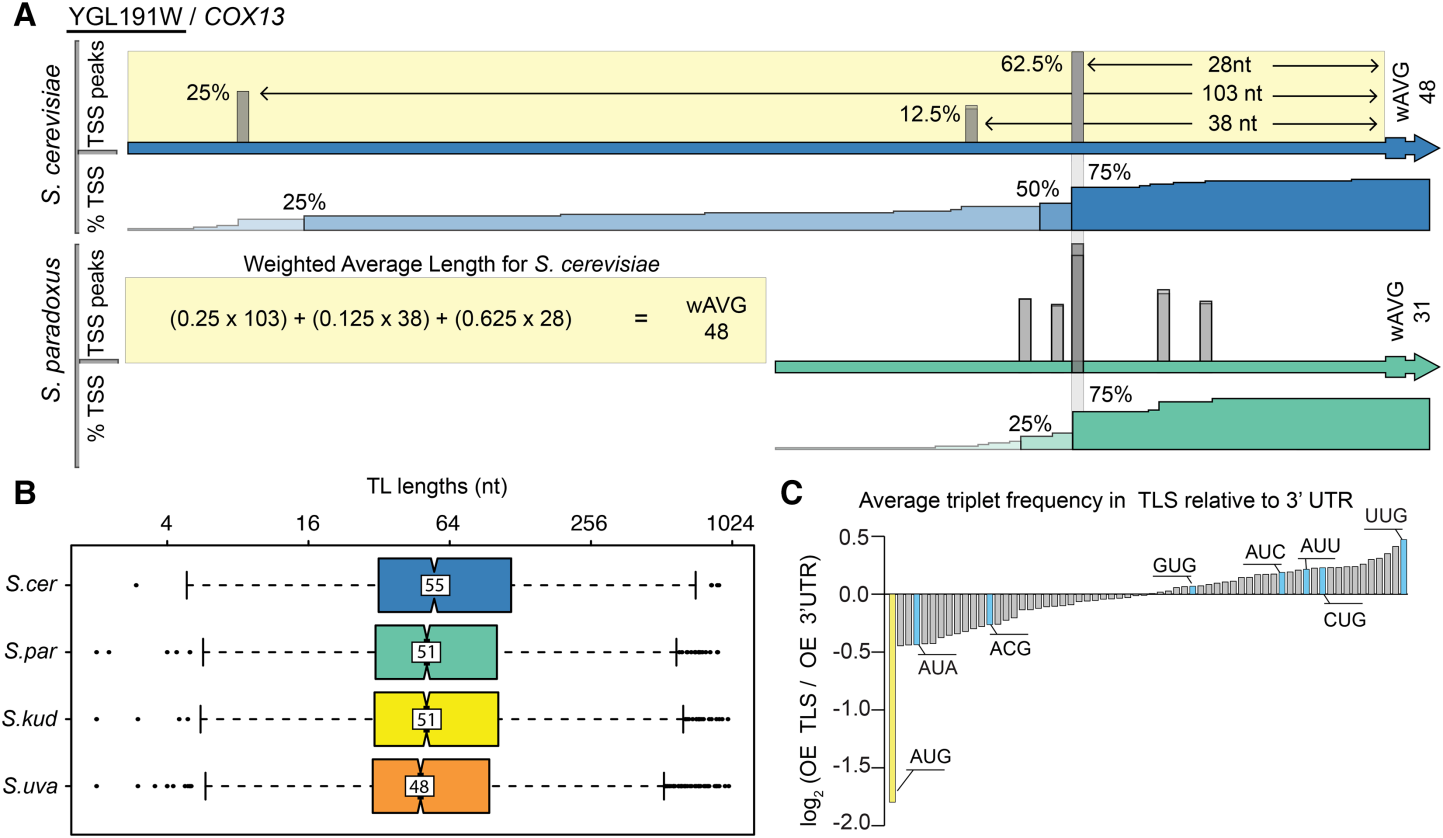
Transcript leader evolution in *Saccharomyces sensu stricto*. (*A*) An example comparison of TLs of *COX13* (YGL191W) from *S. cerevisiae* and *S. paradoxus* is shown. Although the most frequent TSS has been conserved (28 nt TL), the most upstream TSS has diverged. The weighted average (wAVG) length for each transcript leader was calculated to compare transcript leader lengths among species. (*B*) *S. cerevisiae* has significantly longer TLs than the other species. (*C*) The observed/expected ratio (OE) of each 3-nt triplet in 5′ UTRs versus 3′ UTRs is plotted. Relative to the 3′ UTR, the TLSs are de-enriched in AUG triplet nucleotides (black), and enriched in five of seven near-cognate codon (NCC) triplets (gray). The data presented are an average of values from the four species; individual values are plotted in Supplemental Figure S14.

Transcript leader lengths are known to influence translation, with shorter leaders associated with increased translation efficiency (Rojas-Duran and Gilbert 2012). Furthermore, long transcript leaders are more likely to harbor regulatory sequences. We reasoned that this may place evolutionary constraints on transcript leader length, and thus compared transcript leaders among species. Because many genes have multiple transcription start sites, we calculated the weighted average transcript leader length for all homologs in each species (Fig. 2A). Transcript leader lengths were similar in the four species, with medians of 45–55 nts (Fig. 2B). Comparisons between species generally followed their known phylogenetic relationships, with more similarity observed between more closely related species (Supplemental Fig. S1). Transcript leader lengths from our *S. cerevisiae* strain (S288C) were significantly longer than those of other species (Wilcoxon rank-sum test, *P* < 0.029) and had lower correlations with *S. kudriavzevii* and *S. uvarum* than did *S. paradoxus*.

We next calculated leader length coefficients of variation (CV; standard deviation/mean) to compare genes with the slowest and fastest rates of leader length evolution. Genes within the lowest decile of CV values (CV < 0.06) were classified as having the most conserved leader lengths. Of these, short (<55 nt) leaders were found to be enriched in functions involving translation (Gene Ontology [GO] FDR < 0.05) and glucose fermentation (KEGG and Reactome FDR < 0.05), whereas those with long (>55 nt) TLs were enriched in numerous cell signaling and gene regulation GO categories (all at FDR = 0.05) (Supplemental Table S1). These results suggest that selection has maintained short transcript leaders to ensure efficient translation of ribosomal proteins and glycolytic enzymes (Supplemental Fig. S2), and long transcript leaders to allow translational regulation of genes involved in regulating gene expression.

In addition to length, transcript leader sequences can also play a role in gene expression by attracting ribosomes to upstream translation initiation sites. Previous work reported that AUG triplets are depleted from mammalian transcript leaders, yet they are also highly conserved (Churbanov et al. 2005). Although similar results were reported in yeast, only about 250 genes had annotated transcript leaders at the time. We examined enrichment of AUG and NCC triplets in TLSs relative to their frequency in 3′ UTRs (Fig. 2C). As expected, AUG codons were depleted from TLSs, yet were highly conserved between species (Supplemental Table S2). AUG was less depleted in our *S. cerevisiae* (S288C) strain, suggesting that this laboratory strain may have accumulated more upstream AUG (Supplemental Fig. S3). Notably, five of the seven NCC codons are enriched in the TLS relative to the 3′ UTR, with UUG showing the highest enrichment (Fig. 2C). These results suggest that upstream AUG and NCC triplets evolve under different selective pressures, with NCC triplets lacking the strong signal of negative selection associated with upstream AUGs.

### Identification and testing of AUG and NCC uORFs with uORF-seqr

**Figure 3.**
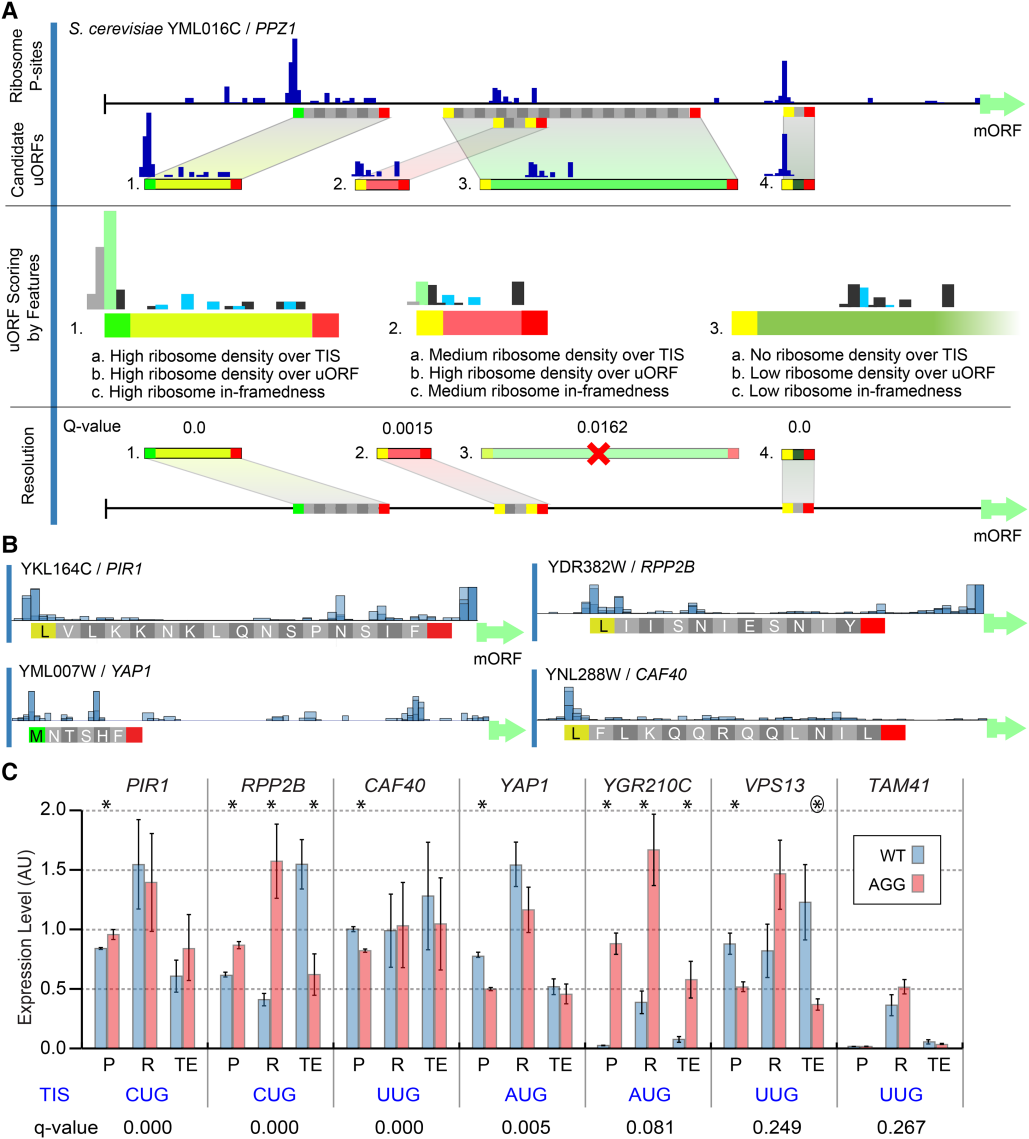
uORF identification using uORF-seqr. (*A*) uORF-seqr relies on genome annotation, nucleotide sequence, and ribosome profiling and RNA-seq data. *PPZ1* (YML016C) is shown as an example. Candidate uORFs (containing potential start and stop codons) are scored for eighteen features (Supplemental Methods) related to Ribo-seq data (P-sites) and uORF position within transcript leaders. A linear regression is used to score each uORF according to the weighted value of these features (Methods), removing candidates that do not score significantly better than null models (Resolution). For overlapping or nested uORFs (e.g., uORFs 2 and 3), the highest scoring candidate uORF is retained. (*B*) P-site fraction data around four predicted uORFs. Although the Lysine codon is shown, translation is expected to initiate with methionine at NCC codons. (*C*) Wild-type (AUG or NCC) and AGG start codon mutants were tested for protein (P) and mRNA (R) levels using a dual fluorescence reporter and qPCR, respectively, in triplicate. Bar graphs show the mean and standard error. Estimated translation efficiencies (TE = P/R) were also compared. Significant differences: (*) *t*-test *P* < 0.05; (circled asterisk) *P* = 0.0549.

Previous studies predicted thousands of AUG and NCC uORFs in *S. cerevisiae* by applying “rule-of-thumb” analyses to Ribo-seq data (Ingolia et al. 2009; Brar et al. 2011). However, these heuristic approaches lack statistical control and are complicated by the limited sequence requirements for uORFs and the potential for confounding artifacts in Ribo-seq data. To identify statistically significant uORFs, we developed uORF-seqr, a novel machine learning approach that uses regression to select and weight features that correlate with uORF detection in biological replicate Ribo-seq data sets (Fig. 3A,B). Importantly, the type of start codon was not taken into consideration to avoid AUG/NCC training bias. Transcript leaders are first scanned for AUG and NCC start codons with in-frame stops. Candidate uORFs are scored for 18 features that capture different aspects of Ribo-seq data and uORF location within transcript leaders (Supplemental Fig. S4). uORF-seqr then trains a regression model based on the number of times each uORF is observed among biological replicates and scores candidate uORFs accordingly. Statistical control is achieved by comparing scores to those produced by a randomized null model (Methods).

We applied uORF-seqr to Ribo-seq data from *S. cerevisiae* and *S. paradoxus* (McManus et al. 2014), as well as new data from *S. uvarum*. Controlling the FDR at β = 0.05, we observed 982 uORFs in 791 *S. cerevisiae* genes, of which a substantial number overlap with predictions previously reported (Supplemental Table S3). Substantially more uORFs were detected in *S. paradoxus* and *S. uvarum*, 1619 and 1672, respectively. Detection is a complex phenomenon, affected by multiple factors (experimental and mathematical), which may account in part for these differences (Discussion). Overall, uORF-seqr identified more statistically significant NCC uORFs than AUG uORFs in each species.

We tested seven *S. cerevisiae* uORF-seqr predictions using a dual fluorescence (YFP/mCherry) reporter plasmid. uORFs were selected across a range of *Q*-values above and below the 5% FDR cutoff (Fig. 3C). For each uORF, we compared levels of protein (YFP/mCherry) and RNA (qPCR) from WT and nonfunctional “AGG” start codon (Kolitz et al. 2009) mutant plasmids (ΔTIS) (Fig. 3C). For six of the seven, start codon mutations significantly changed protein levels. In *RPP2B*, *YAP1*, *YGR210C*, and *VPS13*, mRNA levels also changed significantly, suggesting that uORF presence may affect RNA stability. The largest change in protein and mRNA levels were observed for the AUG uORF found at *YGR210C*. Most of the non-AUG uORFs had smaller, although significant, effects on reporter expression. Importantly, the uORF predicted at the highest FDR (*TAM41*) was the only one that failed to affect reporter expression. These results indicate that uORF-seqr identifies functional uORFs and suggest our 5% FDR is conservative, as two predictions above this threshold also impacted gene expression.

We also evaluated the impact of cycloheximide on uORF-seqr predictions, as recent work indicates it can alter the locations of Ribo-seq footprints (Gerashchenko and Gladyshev 2014; Hussmann et al. 2015). We compared uORF-seqr predictions from a matched data set with and without cycloheximide (Nedialkova and Leidel 2015). uORF-seqr predicted similar numbers of NCC uORFs between the treated and untreated data sets, but AUG uORF identification was limited in the absence of cycloheximide. Indeed, uORF-seqr was unable to correctly identify three of the five known uORFs in *GCN4* when cycloheximide was omitted (Supplemental Fig. S5).

### Comparison of AUG and NCC uORF functional characteristics

**Figure 4.**
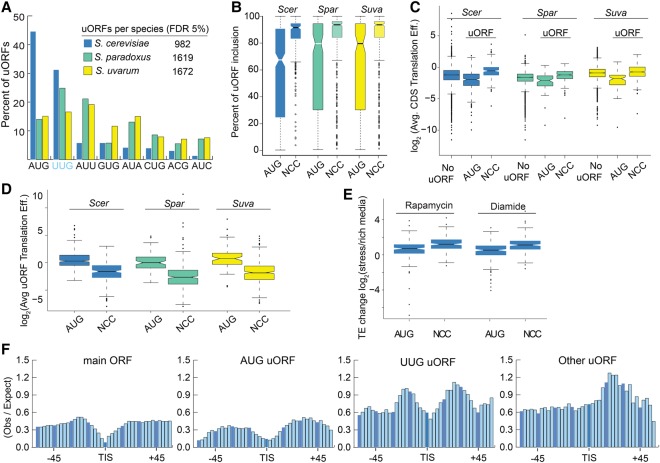
Comparing AUG and NCC uORFs in *Saccharomyces* yeasts. (*A*) In *S. cerevisiae*, nearly 45% of uORFs initiate with AUG, compared with ∼15% in *S. paradoxus* and *S. uvarum*. (*B*) AUG uORFs had significantly lower rates of transcript inclusion (the fraction of a gene's transcripts that contain the uORF; AUG median 73%) compared to NCCs (92%), suggesting alternative transcription initiation plays a greater role in AUG uORF regulation than NCC type. (*C*) Genes with AUG uORFs have lower translation efficiencies, whereas genes with NCC uORFs have higher translation efficiencies than genes without uORFs. (*D*) AUG uORFs have higher translation efficiencies than NCC uORFs. (*E*) The translation efficiencies of AUG and NCC uORFs increase with stress, and NCC uORFs increase more. (*F*) A metagene analysis of RBP interactions around main ORF and uORF start codons is shown using public data (Freeberg et al. 2013). AUG and UUG uORFs have similar patterns of protein occupancy with peaks of occupancy upstream of and downstream from the start codon. This pattern was not observed for other NCC uORFs, which only exhibited a significant peak downstream from the TIS.

Evaluating uORFs among the three species, we identified several general trends. First, AUG-type uORFs make up nearly 45% of all uORFs identified in *S. cerevisiae* but only account for ∼15% in the other two species (Fig. 4A). This is consistent with the increased frequency of upstream AUG we found in *S. cerevisiae* transcript leaders. Second, NCC uORFs are also distinct from AUGs in their rate of inclusion in transcript isoforms (Fig. 4B). AUG uORFs are included far less frequently in transcript isoforms than NCC types in all three species. This suggests that the presence of AUG uORFs may be more dependent on transcription initiation sites. The median distribution of transcript length was 17% longer for genes with uORFs than genes without uORFs, although no general difference in length was observed between genes with AUG-uORFs and NCC-uORFs (Supplemental Fig. S6). Kozak sequence contexts for AUG and NCC uORFs were also similar, as both groups had essentially no consensus sequence around their start codons (Supplemental Fig. S7).

We next investigated the translation efficiencies of uORFs and their corresponding main ORFs (Fig. 4C). Genes with AUG uORFs had significantly lower translation efficiencies than genes without uORFs (WRST, adjusted *P*-value <0.05), which is consistent with their general role as translational repressors (Ingolia et al. 2009; Hinnebusch et al. 2016). In contrast, genes with NCC-uORFs had significantly higher translation efficiencies than genes without uORFs, (WRST, adjusted *P*-value <0.05). This higher translation efficiency may reflect an increased probability of detecting relatively rare events on genes that are highly translated. Indeed, AUG uORFs were more occupied than NCC uORFs (Fig. 4D), consistent with their known higher rates of translation initiation (Kolitz et al. 2009). Finally, we found that both AUG and NCC uORFs had higher translation efficiency under two stress conditions (rapamycin and diamide treatment) (Fig. 4E; Nedialkova and Leidel 2015). These results suggest most NCC uORFs are less well-translated under the tested growth conditions, yet are identified by uORF-seqr in the transcript leaders of highly translated genes due to a high flux of ribosomes.

Previous research identified several RNA binding proteins that regulated uORFs (Ruiz-Echevarría and Peltz 2000; Medenbach et al. 2011; Schleich et al. 2014; Zhang et al. 2017). We investigated protein occupancy around the translation initiation site (TIS) of uORFs using genome-wide cross-linked protein occupancy data (gPAR-CLIP) (Fig. 4F; Freeberg et al. 2013; Methods). We found similar patterns of protein occupancy around main ORF, AUG, and UUG uORF TISs, characterized by decreased occupancy around TISs with peaks upstream and downstream. In contrast, other NCC TISs featured only a downstream peak. Interestingly, AUG uORFs and main ORFs had significantly lower protein occupancy than that observed for the NCC types. These results suggest proteins exert stronger control over NCC uORFs.

### UUG and AUG uORFs have similar rates of conservation

We next examined uORFs found in multiple species. We asked whether genes for which uORFs were detected in all three species were more frequently functionally related than to be expected by chance. The 254 genes with significant uORFs in all three species were enriched for GO terms associated with signaling, cell wall organization, negative regulation of translation, dephosphorylation, and cation transport (adjusted *P*-value <0.05). These are consistent with uORFs functioning in genes involved in the cell-cycle and stress responses.

**Figure 5.**
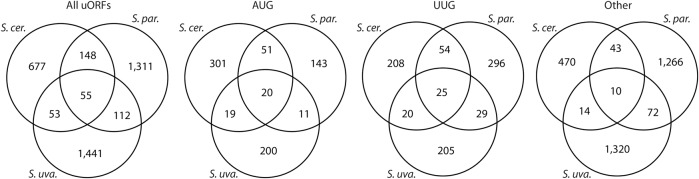
Comparison of statistically significant uORFs in three *Saccharomyces* yeast species. Venn diagrams show the numbers of sequence-conserved (Methods) uORFs identified in each species and the number shared by multiple species, separated by start codon type. The number of shared uORFs generally follows phylogenetic relationships. uORFs initiating with TTG are shared by two or more species at rates comparable to AUG-uORFs, whereas other NCC uORFs are less commonly shared. Note that uORF start codon types were defined by the start codon used in *S. cerevisiae* or *S. paradoxus*. See Supplemental Figures S8 and S11 for a full breakdown of uORF orthologs.

We next considered uORF conservation (presence in at least two species). In principle, the minimal sequence requirements for uORFs permit two modes of conservation. uORFs can be conserved in sequence (“sequence homologs”) or merely in their position within transcript leaders (“position homologs”) (Methods; Supplemental Fig. S8A; Supplemental Table S4). We observed 313 pairs (e.g., a uORF in two species), and 55 triplets, of sequence homologs (Fig. 5). Notably, the fraction of uORFs shared between two or more species was similar for UUG (15.3%) and AUG uORFs (13.6%). In contrast, other NCC uORFs were shared much less frequently (4.4%). These results suggest that AUG and UUG uORFs are conserved at similar rates.

We categorized uORFs based on three aspects: conservation, TL-inclusion, and TIS type (Supplemental Fig. S8B). Genes that had sequence or positional homologs were significantly enriched in functions associated with cell wall organization and biogenesis, relative to all genes containing uORFs (adjusted *P*-value <0.05) (Supplemental Table S5). No similar enrichment was observed for genes with novel uORFs. Considering all uORFs, most were included in the majority of transcript isoforms, with only ∼19% of uORFs being in the minority of isoforms. Interestingly, novel uORFs were more commonly found in alternative regions of transcript leaders. Moreover, uORFs found in the minority of transcript leaders were enriched in AUG uORFs (*S. cerevisiae*: 2.4-fold; *S. paradoxus*: 3.0-fold; *S. uvarum*: 2.5-fold; adjusted *P*-value <0.05, FET), consistent with our earlier observation of decreased rates of AUG uORF inclusion. Overall, the association of uORFs with different mORF-TEs and isoform inclusion rates was similar for conserved and nonconserved uORFs (Supplemental Fig. S9).

## Discussion

Using ribosome profiling, TL-seq, and a novel machine learning algorithm called uORF-seqr, we identified 3334 statistically significant candidate NCC uORFs across three species of yeast. Several aspects of NCC uORFs differ from AUG uORFs, such as higher transcript inclusion frequency and protein occupancy downstream from start codons. Furthermore, we find 788 uORFs are conserved either in sequence or position (Supplemental Table S7). Notably, UUG uORFs are conserved at a rate similar to that of AUG uORFs. Such high rates of UUG uORF conservation suggest they are being maintained over evolutionary timescales, potentially due to regulatory roles. Using publicly available ribosome profiling data, we further found that at least one NCC uORF, in *RPP2B*, is deeply conserved throughout fungi (Supplemental Materials; Supplemental Figs. S8, S10). Together, these results suggest some NCC uORFs have important biological functions.

Several groups have recently applied machine-learning algorithms to mammalian Ribo-seq data to detect AUG uORFs (Fields et al. 2015; Ji et al. 2015; Calviello et al. 2016). Although these studies identified hundreds of uORFs, they did not test them for function or address NCC uORFs. Six of the seven uORF-seqr-predicted uORFs (five NCC and two AUG) we tested affected expression at the RNA and/or protein levels. Notably, the AUG uORF of YGR210C had the largest impact on the reporter, whereas NCC uORFs had smaller effects. This is consistent with expectations, given the weaker recruitment of ribosomes to NCC start codons (Kolitz et al. 2009). The AUG and NCC uORFs we identified appear to function as both positive and negative regulators. Three (one AUG and two UUG) of the uORFs we validated enhanced expression of our reporter construct. Such “enhancer” uORFs have been reported before (Zhang and Dietrich 2005; Waern and Snyder 2013; Andreev et al. 2015) and may allow ribosomes to bypass other, repressive, uORFs downstream (e.g., *GCN4*) (Hinnebusch 2005).

uORF-seqr is data-driven, such that its predictions depend on input data set quality. Data sets with lower 3-nt periodicity may give less reliable results, whereas those with different sequence biases (O'Connor et al. 2016) may lead to different uORF predictions. However, uORF-seqr estimates regression feature weights independently for each set of replicate experiments, which may reduce the impact of data set variability. Indeed, our *S. uvarum* data set has somewhat different sequencing depth and library bias (Supplemental Figs. S12, S13), but relatively similar uORF-seqr models were learned for all three (Supplemental Table S6). Importantly, the general characteristics of *S. uvarum* uORFs were very similar to those of *S. paradoxus* and revealed hundreds of homologous uORFs. In principle, uORF-seqr could be applied to data sets from other species, including metazoans. However, this would require modification to handle genes with alternative splicing of transcript leaders. In addition, uORF-seqr requires three or more biological replicates, and this is uncommon in ribosome profiling data sets.

Yeast express diverse transcript isoforms that may alter the presence of uORFs (Pelechano et al. 2013; Waern and Snyder 2013). Previous comparative genomics of yeast uORFs assumed that entire intergenic regions were transcribed (Cvijović et al. 2007) or that other species use the same transcription start sites found in *S. cerevisiae* (Zhang and Dietrich 2005; Selpi et al. 2009). By annotating the TLs from four *Saccharomyces* species, we defined the “search space” for uORFs in each species. Importantly, AUG uORFs are more common in alternative transcript regions than NCC uORFs. This suggests AUG uORFs are regulated by alternative transcription initiation site choice, whereas NCC uORFs, initiating with weaker start codons, may be regulated by other means (e.g., RNA binding proteins or global changes in translation initiation). Importantly, novel uORFs have much lower rates of inclusion than conserved uORFs, suggesting that uORF innovation may occur predominantly in alternative transcript isoforms. This scenario would facilitate evolutionary “tinkering” with new uORFs by introducing them in transcript leader regions that are conditionally expressed (Jacob 1977; Lareau et al. 2004; Resch et al. 2009).

The correlation of uORF innovation and rates of inclusion is particularly striking regarding the novel AUG uORFs observed in *S. cerevisiae* (Fig. 4). Our previous work showed that differences in upstream AUG frequency between *S. cerevisiae* and *S. paradoxus* were negatively correlated with differences in translation efficiency (McManus et al. 2014). However, the increased transcript leader length and rate of AUG uORFs in *S. cerevisiae* may be specific to the laboratory strain, S288C. S288C has undergone significant evolution during laboratory domestication and has one of the highest rates of upstream AUG compared to other *S. cerevisiae* strains (Supplemental Fig. S3). Interestingly, prior work modeling the evolution of transcript leaders predicted they would increase in length and uAUG frequency in the absence of purifying selection (Lynch et al. 2005). In light of this model, our results suggest that S288C transcript leaders have undergone relaxed selection during laboratory domestication.

Although the AUG and UUG uORFs we identified have similar rates of sequence conservation, there are relatively few deeply conserved uORFs. The relatively modest number of deeply conserved uORFs may reflect the large amount of divergence present in the *Saccharomyces* genus. It has been estimated that protein sequence divergence between *S. uvarum* and either of *S. cerevisiae* or *S. paradoxus* is similar to that found between humans and chickens (Dujon 2006). Thus, uORFs shared by even two species have been maintained over many generations. However, although sharing much of their sequence, some sequence conserved NCC uORFs have divergent start codons (Supplemental Fig. S10). This may in part stem from the difficulty of identifying NCC start codons from Ribo-seq data (e.g., “AUUG” contains two adjacent NCC codons). Alternatively, NCC start codons may be somewhat functionally interchangeable.

We also investigated the potential for protein-mediated regulation of NCC uORFs and found higher protein occupancy immediately downstream from NCC start codons compared with AUG initiating uORFs. We propose that NCC uORFs are more often regulated by RNA binding proteins. In this model, protein binding downstream from NCC start codons may increase uORF translation, similar to what was previously reported for *Sxl* binding downstream from a uORF in *msl-2* (Medenbach et al. 2011). Because most known uORFs function as translation repressors, this would likely lead to down-regulation of the downstream ORFs. There may be much to learn regarding the regulatory roles of proteins on uORF function.

By applying statistical control, validation, and conservation analyses, this study supports the biological significance of some candidate NCC uORFs. Future work is needed to determine the functions of more of these uORFs. Testing large numbers of uORFs will require new high-throughput assays, perhaps similar to those used recently to test transcription factor binding sites (Sharon et al. 2012; Kheradpour et al. 2013). In addition, translation regulation is known to be particularly sensitive to environmental conditions, cell-cycle phases, and developmental transitions. Determining the function of uORFs may thus require sensitive assays of dynamic reporter systems.

## Methods

### Growth of yeast strains, RNA extraction, and mRNA enrichment

Strains (Scannell et al. 2011; McManus et al. 2014) were grown to mid-log phase in 50 mL liquid YEPD media at 30°C (*S. cerevisiae* S288C, *S. paradoxus* CBS432, *S. kudriavzevii* FM1340) or at 24°C (*S. uvarum* JRY9191). Cells were pelleted and resuspended in 1 mL RNA buffer (500 mM NaCl, 200 mM Tris-HCl pH 7.5, 10 mM EDTA). One round of acid-phenol-chloroform extraction and one round of chloroform isoamyl alcohol extraction were performed. The RNA was precipitated with one volume of isopropanol and resuspended in nuclease-free water. Contaminating genomic DNA was removed using the DNA-free Kit (Ambion, Thermo Fisher Scientific). For each TL-seq and poly(A)-mapping library, 100 µg total RNA was enriched for mRNA using Dynabeads Oligo(dT)_25_ beads (Thermo Fisher Scientific) according to the manufacturer's instructions.

### High-throughput sequencing library preparation

5′ and 3′ end mapping libraries were prepared as previously described (Yoon and Brem 2010; Arribere and Gilbert 2013; Supplemental Materials). Using Bowtie (Langmead et al. 2009) default parameters, 5′- and 3′-end reads were aligned to corresponding genomes and converted to single nucleotide read-end pileups. To identify significant read peaks, we simultaneously inferred the size and locations of constant backgrounds using first-order Poisson trend filtering with outlier detection as described (Ramdas and Tibshirani 2016; Supplemental Methods). RNA-seq and Ribo-seq library preparation was performed as previously described (McManus et al. 2014; Spealman et al. 2016), except that *S. uvarum* libraries were produced using the ART-seq kit (Illumina) (for full details, see Supplemental Materials).

### uORF-seqr regression and statistical control

Candidate AUG and NCC uORFs (cuORFs) were identified from transcript leader regions and scored for 18 features related to uORF position within the TLS and ribosome profiles (Supplemental Methods; Supplemental Fig. S3). For each experiment, a regression model was trained to predict the fraction of (biological) replicates, within which each cuORF was detected. These models differed somewhat between experiments to accommodate technical variability of ribosome profiling (Supplemental Methods; Supplemental Table S6).

### Testing uORF functions

A dual fluorescence reporter plasmid was generated by cloning YFP (Metzger et al. 2015) into the vector PTH761 (Chu et al. 2013), such that UTR sequences could be inserted upstream of YFP using XmaI and BglII restriction sites (Supplemental Methods). Transcript leaders were cloned between the *GPM1* transcription start site and YFP. Plasmids were transformed into *S. cerevisiae* (BY4741), and individual colonies were grown in 5 mL URA media to O.D._600_ ∼ 1.0. mCherry and YFP levels were measured using a Tecan M1000. To compare YFP and mCherry mRNA, total RNA was extracted from each culture (as above). qPCR was performed using the SuperScript II Platinum SYBR Green One-Step qRT-PCR kit (Invitrogen) per the manufacturer's instructions (Supplemental Methods).

### Sequence and positional homology identification

To identify sequence homologs, we aligned the nucleotide sequence of candidate uORFs from one species to the TL sequences of homologous genes in the other species using MUSCLE (Edgar 2004). A *Z*-score was generated for each MSA using BLASTZ (Schwartz et al. 2003) score of pairs with HOXD substitution matrix values (Chiaromonte et al. 2002). *Z*-scores were used to break multiple alignment ties. If a candidate uORF alignment overlaps a predicted uORF in the other species with a Jaccard index of 0.6 or greater, they are called sequence homologs. Position homologs are calculated independently for both start and stop codons. The positions of candidate start and stop codons are measured relative to the main ORF. If a uORF start or stop codon is located within 5 nt of this position in another species, those uORFs are called positional homologs. Subsequently, many sequence conserved homologs are also position homologs. Such cases were defined as sequence conserved.

### RBP analysis

Global (gPAR-CLIP) *S. cerevisiae* RBP binding sites were taken from Freeberg et al. (2013). A meta-analysis of protein occupancy was performed by summing sites in 3-nt steps around TISs. Each sum is the “Observed” rate. The background expected protein occupancy rates were determined by random sampling (1000-fold) of TISs and RBP sites. To avoid sampling outside TLs, only genes with a transcript long enough to enclose a given step were included for that step. We excluded the 15-nt region upstream of the main ORF TIS from the TL regions used in uORF analysis to avoid counting main ORF protein interactions as uORF protein interactions.

## Data access

Data from this study have been submitted to the NCBI Sequence Read Archive (SRA; https://www.ncbi.nlm.nih.gov/sra) under accession number SRP109132.

## Competing interest statement

S.K. and L.F. are employees of Illumina, Inc.

## Supplementary Material

Supplemental Material
