# Supplementary material for: Conserved non-AUG uORFs revealed by a novel regression analysis of ribosome profiling data
Source: Genome Res. 2018 Feb;28(2):214–22. doi: 10.1101/gr.221507.117 (PMC5793785; doi:10.1101/gr.221507.117)
Supplement: Supplemental Material [file supp_28_2_214__index.html]

Conserved non-AUG uORFs revealed by a novel regression analysis of ribosome profiling data — Supplemental Material 

# Conserved non-AUG uORFs revealed by a novel regression analysis of ribosome profiling data

## Supplemental Material

- Supplemental\_Files\_1.zip
- Supplemental\_Files\_2.zip
- Supplemental\_Files\_3.zip
- Supplemental\_Files\_4.txt
- Supplemental\_Table\_S1.xlsx
- Supplemental\_Table\_S4.xlsx
- Supplemental\_Table\_S7.xlsx
- Supplemental\_Methods.docx
