# Supplementary material for: Conserved non-AUG uORFs revealed by a novel regression analysis of ribosome profiling data
Source: Genome Res. 2018 Feb;28(2):214–22. doi: 10.1101/gr.221507.117 (PMC5793785; doi:10.1101/gr.221507.117)
Supplement: Supplemental Material [file supp_gr.221507.117_Supplemental_Methods.docx]

### Supplemental methods:

*TL-seq libraries*

TL-seq libraries were prepared as previously described (Arribere and Gilbert 2013). PolyA-enriched mRNA was fragmented using RNA Fragmentation Reagents (Ambion, 2’ at 70°C in 20 μl total), purified using a Zymo RNA Clean & Concentrator™ - 5 column, and eluted in 10 μl of H_2_0. Fragmented RNA was dephosphorylated with 15 units of Antarctic phosphatase (NEB) and 20 units of SUPERase-In (Thermo Fisher Scientific) at 37°C for one hour in a 30 μl reaction in 1 x antarctic phosphatase buffer. Dephosphorylation was stopped by adding EDTA (59 mM final) and incubating at 65°C for 5 minutes. The RNA was purified using an RNA Clean & Concentrator™ column and eluted in 16.75 μl of H_2_0. 5’ mRNA caps were removed using 5U of Tobacco Alkaline Phosphatase in 1X buffer (Epicentre) with SUPERase-In (20U), incubated at 37°C for 2 hours in 20 μl total. The RNA was purified using an RNA Clean & Concentrator column™ and eluted in 10.5 μl of H_2_0. The resulting decapped RNA was ligated to 250 ng of 5′ linker [5’–ACACGACGCT CTTCCGATCT rNrNrNrNrRrRrR]. The 5′ linker was incubated at 70°C for 2 minutes and placed immediately on ice. The RNA and 5′ linker were incubated at 22°C overnight in a 30 μl reaction (10% DMSO, 1X Quick Ligase Buffer (NEB), 20 units SUPERase-In, and 15 units T4 RNA ligase 1 (NEB)). The RNA was purified using a RNA Clean & Concentrator ™ column ( >200 nucleotide protocol, and eluted in 10 μl of nuclease free water. The RNA was then treated with 15 units of T4 PNK (NEB), and 20 units of SUPERase-In at 37°C for 1 hour (20 μl, no ATP) in order to remove 3’ terminal phosphates. The RNA was purified using an RNA Clean & Concentrator ™ column and eluted in 10.75 μl of nuclease free water using the > 200 nt protocol. The RNA was mixed with 375 ng of 3’ linker [/5rApp/TTTAACCGCG AATTCCAG/3ddC/] and heated to 80°C for 2 minutes and placed on ice. The RNA and 3’ linker were ligated at 16°C overnight in a 25 μl reaction (12.5% PEG 8000, 10% DMSO, 20 units SUPERase-In, 200 units T4 RNA Ligase 2 truncated (NEB), and 1X T4 RNA ligase buffer (NEB)) The RNA was purified using a RNA Clean & Concentrator ™ column (>200 nt-long protocol), and resuspended in 11 μL of nuclease free water. The RNA was reverse transcribed using Superscript® III Reverse Transcriptase (Thermo Fisher Scientific) at 50°C for 30 minutes according to the manufacturer's instructions, using the reverse transcription primer [5’-GTGACTGGAG TTCAGACGTG TGCTCTTCCG ATCCTGGAAT TCGCGGTTAA A]. The RNA template was destroyed with NaOH (100 mM final) incubated at 98°C for 20 minutes. The cDNA was ethanol precipitated and resuspended in 20 μl TE. cDNA (~150 to 330 nt) were extracted from 8% polyacrylamide 8M urea gels and resuspended in 10 μl of TE. cDNA were PCR amplified to add Illumina sequences (universal fwd [5’–AATGATACGG CGACCACCGA GATCTACACT CTTTCCCTAC ACGACGCTCT TCCGATCT], illumina indexed reverse primers [5′- CAAGCAGAAGA CGGCATACGA GAT -(6 nt Truseq index)- GTGACTGGAG TTCAGACGTG TGC]. In a 20 μl reaction, 2 μl of cDNA template, 0.5 μM each forward and reverse primer, 0.5 mM dNTPs, 0.4 units Phusion ® HiFi DNA polymerase (NEB), and 1X Phusion HF buffer, were cycled 18 rounds (55°C annealing). Libraries were purified using a Zymo DNA Clean & Concentrator™ column, and eluted in 10 μl of H_2_0. Library size and concentration was assessed by Agilent TapeStation analysis.

*3’- end mapping sequencing libraries*

PolyA-enriched mRNA was fragmented (Ambion Fragmenation Reagents, 3’ at 70°C in 20 μl total), purified using an RNA Clean & Concentrator™column, and eluted in 11 μl of nuclease free water. The RNA was reverse transcribed using Superscript® III Reverse Transcriptase at 50°C for 30 minutes according to the manufacturer's instructions. The reverse transcription primer [/5Phos/AGATCGGAAG AGCGTCGTGT AGGGAAAGAG TGTAGATCTC GGTGGTCGC/iSp18/C ACTCA/iSp18/TTCAG ACGTGTGCTC TTCCGATCTT TTTTTTTTTT TTTTTTTTVN–3’] is complementary to poly A and contains sequence complementary to Illumina forward and reverse PCR primers. After reverse transcription, the RNA template was destroyed by adding NaOH (100 mM final) and incubating at 98°C for 20 min. The cDNA was ethanol precipitated and resuspended in 20 μl TE. Fragments (130 to 140 nt) were extracted from 8% polyacrylamide 8M urea gels and resuspended in 7.5 μl of TE. cDNA fragments were circularized with 50 units CircLigase ssDNA Ligase (Epicentre) at 60°C for 1.5 h according to the manufacturer's instructions. The circularized cDNA was amplified for 12 cycles as previously described (Talkish et al 2014). DNA fragments (~170 bp) were extracted from a native 6.5% acrylamide TBE gel and resuspended in 10 μl of TE. The purified PCR products were subjected to 18 more cycles of PCR (as previously described) using 2 μl of purified PCR product as template for the second round of PCR. The libraries were purified using a DNA Clean and Concentrator™ column, and eluted in 11 μl of nuclease free water. The libraries were assessed and quantified using an Agilent 2200 TapeStation System.

*Significant TSS and pA sites identification*

5’- and 3’-end mapping reads were aligned to corresponding genomes using bowtie (default parameters) and converted to single nucleotide read-end pileups. Aligned read peaks (TSS var. pA) were assumed to derive from two additive processes: a background rate (proportional to expression level and local sequence bias) and whether the peak was a true TSS or pA site. To deconvolve these, we simultaneously inferred the size and locations of constant backgrounds first-order Poisson trend filtering with outlier detection as described (Ramdas and Tibshirani, 2016). In this case, outliers are peaks above background. Briefly, the Poisson trend filtering model asserts a (convex) penalty for sequential nucleotides having different Poisson rates; increasing penalization encourages contiguous regions of constant Poisson background rate. These regions are determined entirely by the degree of penalization and the aligned read distributions. Outlier detection is a function of a second penalization term which limits the total outlier mass. These two penalty terms were identified stagewise using the Gap statistic (Tibshirani et al., 2001): first, a no-outlier model was fit relative to 11 reference distributions, for which we permuted the locations of pileups along each chromosome. Values of the penalty term for an oversmoothed and an undersmoothed model were identified as the first sign change of the gap statistic from negative to positive, respectively. We then relaxed the outlier penalization term to move from the gap statistics of the undersmoothed model to those of the oversmoothed model, at which point all large “above background” peaks would no longer influence the mean Poisson read rate, for regions roughly scaled as in the oversmoothed model. P-values were calculated for each above-background peak relative to the mean estimated background read rate of the region it belonged to; these taken together were FDR controlled by the Benjamini-Hochberg procedure. The same method was separately applied to the pA reads to identify plausible polyadenylation sites.

*RNA-seq and Ribosome Profiling*

Published ribosome profiling and matched RNA-seq data (two replicates) for *S. cerevisiae* and *S. paradoxus* were used (McManus et al. 2014; SRA #SRP028552). A third replicate from cells grown in a different batch of media was prepared as previously described (McManus et al. 2014; Spealman et al. 2016), as uORF-seqr requires triplicate data. Ribosome profiling and RNA-seq libraries for *S. uvarum* were prepared using the yeast ARTseq ribosome profiling kit (Illumina, Inc) following the manufacturer’s protocol. The use of this protocol has a minor effect on the footprints (Supplemental Figure S11). Library reads were aligned with STAR (Dobin et al. 2013) allowing one mismatch. Reads exceeding ten mappings were discarded, and intron alignment was suppressed (max intron length 1nt). Ribosome P-sites were estimated for reads whose genome aligned lengths (i.e. after excluding “soft match” prefix and suffix positions) ranged from 27 to 33 nucleotides. The P-site corresponding to 28 nucleotide matches was placed +12nt from the read 5’-end (Ingolia et al. 2009). For other lengths, each position between +12nt from the 5’-end to -19 from the 3’-end of a read was considered equally probable, and assigned an equal fraction of a read for a P-site.

*uORF-seqr regression and statistical control*

Candidate AUG and NCC uORFs (cuORFs) were identified from transcript leader regions and scored for eighteen features related to uORF position within the TLS and ribosome profiles (Supplemental methods, Fig. S3). For each experiment, a model was trained to predict the fraction of (biological) replicates within which each cuORF was detected. These models differed between experiments in order to accommodate technical variability of ribosome profiling. The subset (T) of cuORFs that did not overlap with any other cuORF was identified. These were used as training data to avoid multiple-counting of ribosomes in different reading frames/cuORFs. A L1-penalized sparse binomial regression (generalized linear model with a log-linkage; R glmpath package) was fit by 10-fold stratified cross-validation over the set T to determine the L1 penalization parameter by minimizing holdout squared error. Stratification was performed over genes (all cuORFs of the same gene were either all held-out or held-in for any fold). The final regression used the minimizing penalization parameter and was fit to all of the cuORFs in T. Each cuORF was assessed with this regression (including those not in T) in turn. Statistical control was asserted on cuORFs by comparison to regressions trained with randomly permuted ribosome protection data null and to a reference distribution made by column-independent permutation of the feature vectors of the observed cuORFs.

*uORF-seqr regression feature definition*

Candidate uORFs (cuORFs) initiating with AUG and NCC start codons and terminating with in-frame stop codons were identified from transcript leader regions and scored for 18 features related to uORF position within the TLS and ribosome profiles. Ribosome profile features included those derived from a Discrete Fourier Transform (DFT; derived using numpy.fft) taken over the ribosome profile of a cuORF. The features extracted from each cuORF Fourier transformation describe those related to amplitude (in units of P site ribosome occupancy): (a) the maximum amplitude of any nontrivial frequency (i.e. the maximum proportion of P site ribosome occupancies associated to a particular nucleotide step size) as well as (b) the phase of the corresponding stride (in radians; capturing the relative offset of the stride from the start codon of the cuORF); (c) the maximum amplitude and (d) phase of the within-frame frequency (analogously defined as for (a,b)). Since frequency is not in units of nucleotides, we included a feature (e) to capture the nucleotide spacing of the frequency of (a,b). To normalize for expression level differences among replicate libraries, copies of cuORF features (a,c) were divided by the fraction of total ribo-seq P site coverage of the corresponding TLS out of the total in the replicate library to form features (f) and (g), respectively (unitless features). By the same normalization, we included (h) the normalized median amplitude (across nontrivial frequencies). Features (i,j) captured the normalized (as above) sum of P site coverage in the TLS upstream and downstream of the cuORF, respectively. A similar DFT was performed on the whole of the TLS, and (k) the maximum amplitude of any nontrivial frequency (analogous to feature (f)) was also recorded; this feature was the same for all cuORFs in the same TLS. We also calculated (l) a “relative start magnitude” (Brar et al. 2011) of each cuORF: the number of P sites at the first nucleotide of the start codon minus the average P site coverage of all nucleotides two codons upstream, normalized as above. Normalization enabled comparison of features across libraries with differing read depths. “Positional” features were (m) the cuORF length; (n) distance (in nt) between the TSS and cuORF start, (o) distance between the cuORF end and main ORF start; (p,q) whether the cuORF starts (resp. ends) within a possible N-terminal extension region of the main ORF. The possible N-terminal extension region spans the closest in-frame (relative to the main ORF TIS) upstream stop codon to the main ORF TIS. The final positional feature was (r) the fraction of the length of the cuORF that does not overlap the main ORF. See Table S6 for a comparison of feature weights learned from different datasets.

*Dual fluorescence assay for uORF validation*

­­YFP was amplified from *S. cerevisiae* YPW1784 genomic DNA (Metzger et al., 2015), using primers that introduced sites for XmaI upstream of YFP, BglII through a silent mutation in the second codon of YFP, and NotI downstream of YFP (see Supplemental Methods). The YFP forward primer also introduced a silent mutation in YFP to create a BglII site to allow inserting transcript leaders without introducing additional sequences. The primers, YFP-XmaI-BglII-F 5’-AAACCCGGGA ATAAACACAC ATAAACAAAC AAAATGAGAT CTAAAGGTGA AGAATTATTC ACT-3’ and YFP-NotI-R 5’-GGAATTGCGG CCGCTGTTACAT GCGTACACGC GTTTG-3’) were used to amplify YFP while also incorporating XmaI and NotI sites. The YFP PCR product was digested with XmaI and NotI and cloned into the vector PTH761 (Chu et al., 2013). The *GPM1* promotor was amplified from *S. cerevisiae* genomic DNA with primers GPM1-SalI-F 5’-GATGTCGACG GAAAGATACT AGCGCGCGC and GMP1-XmaI-R 5’-GTACCCGGGG TAATATGTGT GTTTGTTTGG ATTA-3’, also incorporating SalI and XmaI. The GPM1 PCR product was then digested with SalI and XmaI, and cloned into the PTH761 vector upstream of YFP. Transcript leaders were cloned between the *GPM1* promoter and YFP using XmaI and BglII restriction sites. The resulting plasmids were then transformed into the *S. cerevisiae* strain BY4741. Positive colonies were selected on minimal media lacking uracil. Protein levels were measured by growing individual colonies in 5ml of URA- media to an O.D._600_ of approximately 1.0. mCherry and YFP levels were measured using a Tecan M1000 at 587 ± 5nm and 610 ± 5nm excitation and emission for mCherry and 514 ± 5nm and 527 ± 5nm for YFP. In parallel, total RNA was extracted from the cultures using acid phenol chloroform (described above). Total RNA was treated with DNAse I to remove genomic DNA. To compare YFP and mCherry mRNA levels, quantitiative PCR was performed using the SuperScript® II Platinum® SYBR® Green One-Step qRT-PCR kit (Invitrogen) according to the manufacturers instructions using primers YFP-QPCR-F 5’- GGTTGAATTA GATGGTGATG TTAATGG-3’, YFP-QPCR-R 5’-GCATCACCTT CACCTTCACC GGAGACAG-3’, mCherry-QPCR-F 5’- TTTTGCATGA CTGGACCATC AGATG-3’, and mCherry-QPCR-R 5’- CATTTACAAG GTCAAGTTGA GAGGTACC-3’ producing 66 bp and 60 bp amplicons for YFP and mCherry, respectively.

*RPP2B uORF TIS analyses*

We aligned the RPP2B TLS (~100nt upstream region) of 30 species of fungi using Jalview (Waterhouse et al. 2009) and MUSCLE (Edgar 2004) with default settings (Supplemental File 8). In cases where paralogs within a species had not been

distinguished we used the more conserved paralog (Supplemental File 9). Manual realignments and trimming were performed using Jalview (Supplemental File 10,11). Trimmed alignments are depicted in Figure S7. Species lacking an alignment to the proposed motif were removed for the generation of the logo figure, the logo figure was generated using Weblogo (Crooks et al. 2004). We manually evaluated public ribosome profiling data for ribosome occupancy on the uORF TIS in three fungal species (*Candida albicans*: GSE52236, *Schizosaccharomyces pombe*: E-MTAB-2179, *Neurospora crassa*: GSE71032).

### Additional Supplemental Material

### Supplemental Files 1. Re-annotated Genomes.

File A. Name: saccharomyces_paradoxus.gff

Contents: Re-annotated *Saccharomyces paradoxus* genome

File B. Name: saccharomyces_kudriavzevii.gff

Contents: Re-annotated *S. kudriavzevii* genome

File C. Name: saccharomyces_bayanus_inc_GCN4.gff

Contents: Re-annotated *S. uvarum* (previously *S. bayanus*.) genome

File D. Name: saccharomyces_cerevisiae.gff

Contents: Annotation of *S. cerevisiae* genome used in this experiment.

### Supplemental Files 2. Predicted uORFs with q-values.

File A. Name: saccharomyces_cerevisiae-uorfs.bed

Contents: Annotated significant *S. cerevisiae* uORFs with q-values

File B. Name: saccharomyces_paradoxus-uorfs.bed

Contents: Annotated significant *S. paradoxus* uORFs with q-values

File C. Name: saccharomyces_uvarum-uorfs.bed

Contents: Annotated significant *S. uvarum* uORFs with q-values

**Supplemental Files 3. Rpp2b Multiple sequence trimmed alignment.**

File A. Name: Rpp2B_Fungi.txt

Contents: TL sequences from 33 different loci from 30 fungal species before alignment, with comments and Accessions.

File B. Name: RPP2B_all_untrimmed.mfa

Contents: MSA alignment of RPP2B nucleotides from 33 different loci from 30 fungal species. Homologs have not been resolved and have are denoted with a ‘_#’ suffix.

File C. Name: RPP2B_aligned_untrimmed.mfa

Contents: MSA alignment of RPP2B nucleotides from 30 fungal species before trimming and homolog resolution. Homologs were resolved by taking the highest scoring pair.

File D. Name: RPP2B_aligned_trimmed.mfa

Contents: Manually trimmed MSA alignment of RPP2B nucleotides from 30 fungal species, with comments and Accessions.

**Supplemental Files 4. Triplet nucleotide conservation in TLs**

Contents: Sum of all triplet nucleotides within the TLs of S. cerevisiae, S. paradoxus, and S. uvarum. Number of conserved triplets within pairwise alignments of each species is also reported.

**Supplemental Figures:**


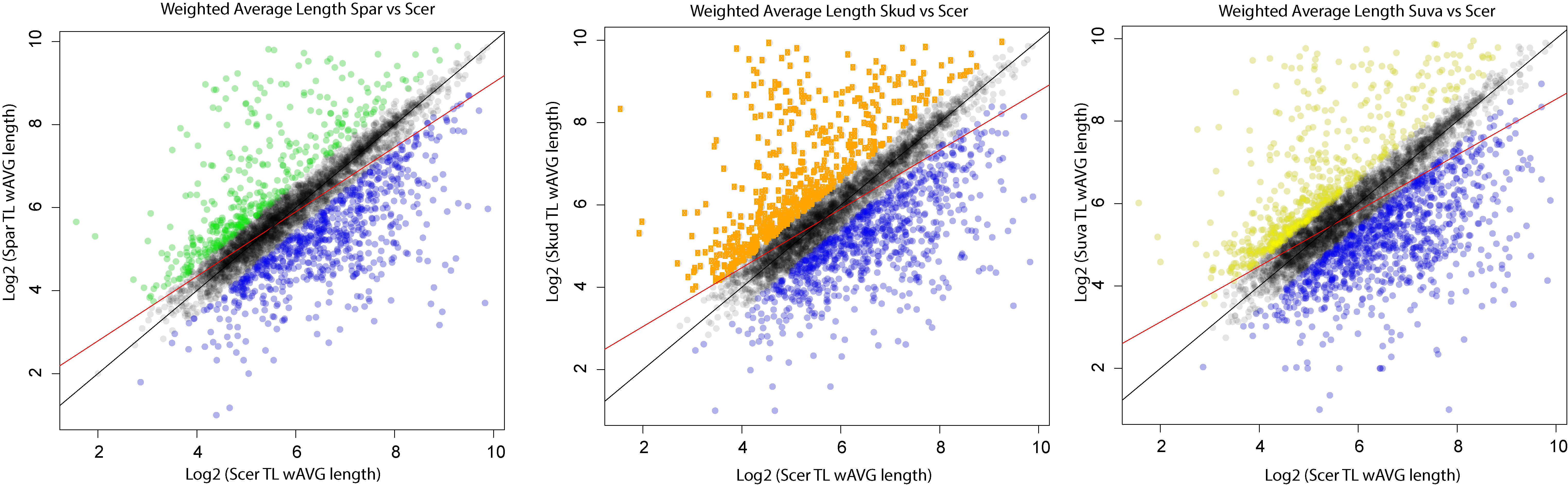


### Figure S1. Weighted average length of TLs between species. Comparisons of weighted average (wAVG) transcript leader lengths are shown. We find decreasing correlation (R^2^) in TL lengths with increasing evolutionary distance.


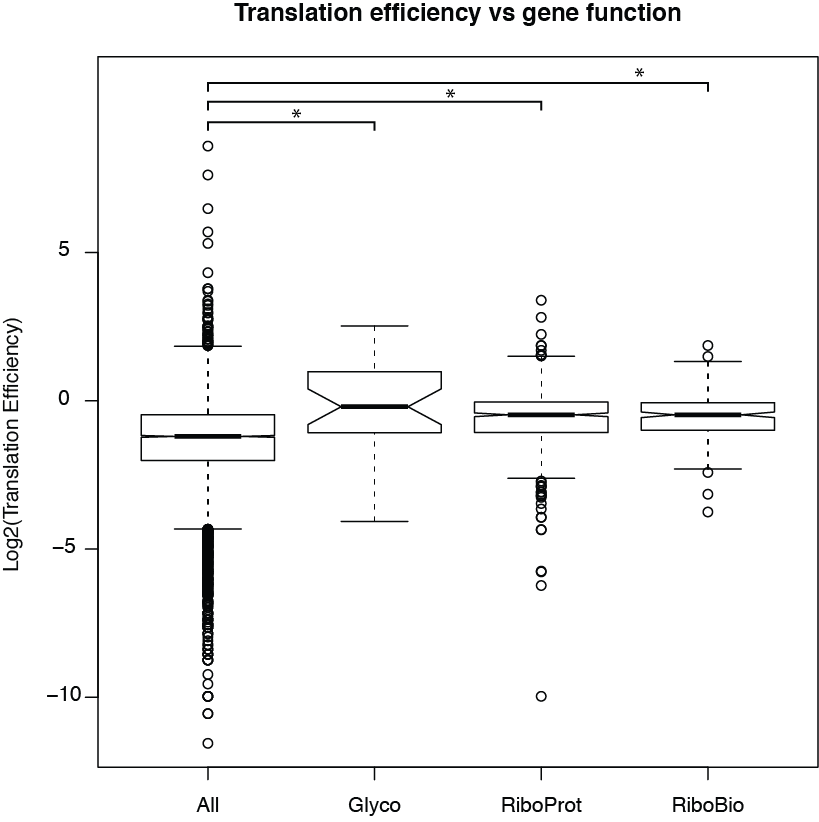


### Figure S2. Ribosomal proteins and glycolytic enzymes are translated more efficiently than other genes. The average translation efficiency of all *S. cerevisiae* genes, genes encoding glycolytic enzymes (Glyco), ribosomal proteins (RiboProt) and ribosome biogenesis factors (RiboBio) are plotted. Ribosomal proteins have a 1.7-fold higher median translation efficiency, while glycolytic enzymes have a 2-fold higher translation efficiency. Asterisks indicate P < 0.001, Wilcoxon rank-sum test.

##
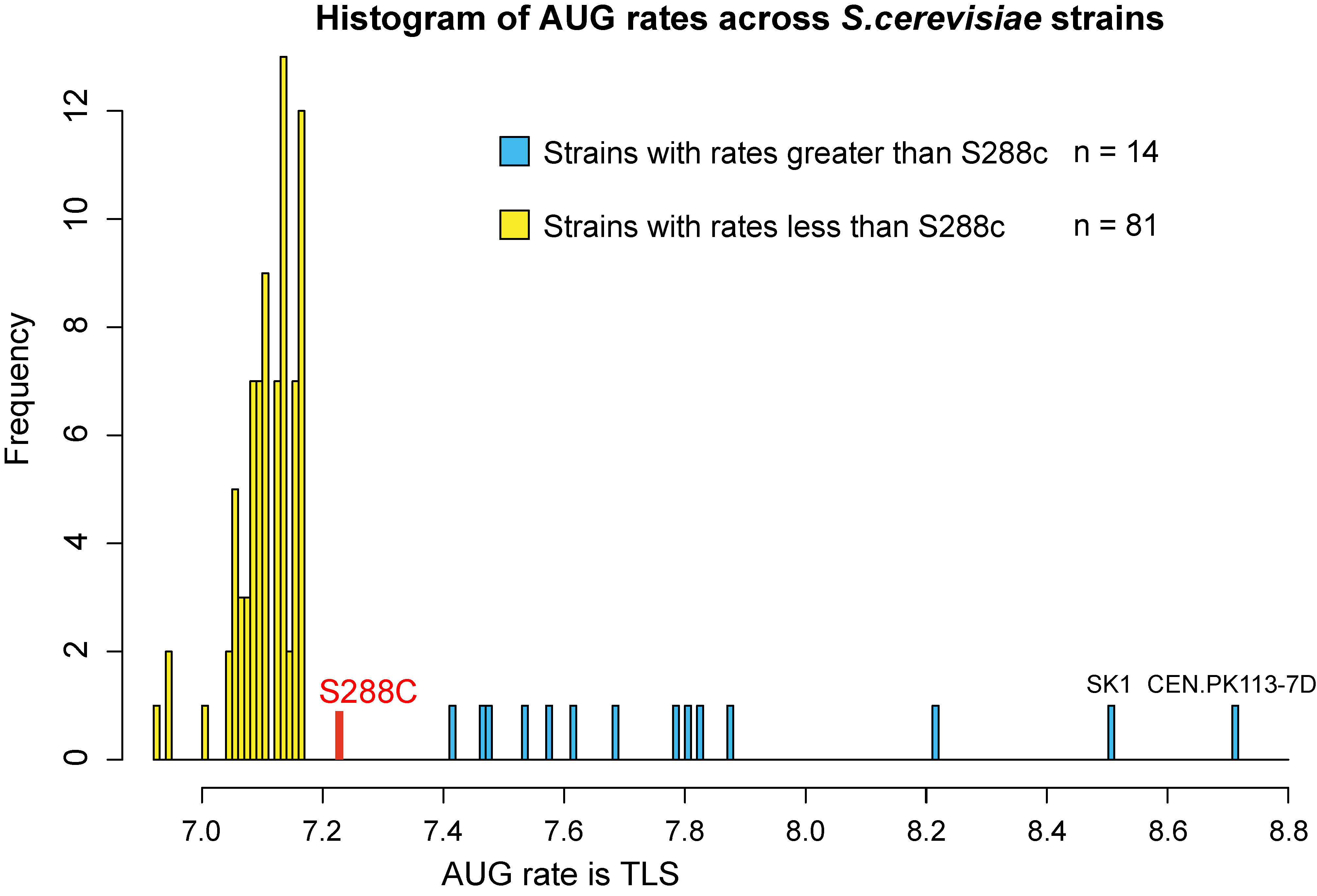


### Figure S3. Comparison of AUG rates across strains. To determine if AUG frequencies in *S. cerevisiae* were the result of species or strain-specific enrichment we counted AUGs in 95 additional *S. cerevisiae* strains (Strope 2015) using our TLS annotations from the S288C lab strain. The rates of AUG identified in S288C were higher than 87% of other sequenced yeast strains. Two additional lab strains Cen.PK and SK1 were also included in this analysis; these had AUG rates similar to, but elevated above, S288c. Notably, this analysis assumes that all the strains have transcript leader lengths equal to those of S288C.


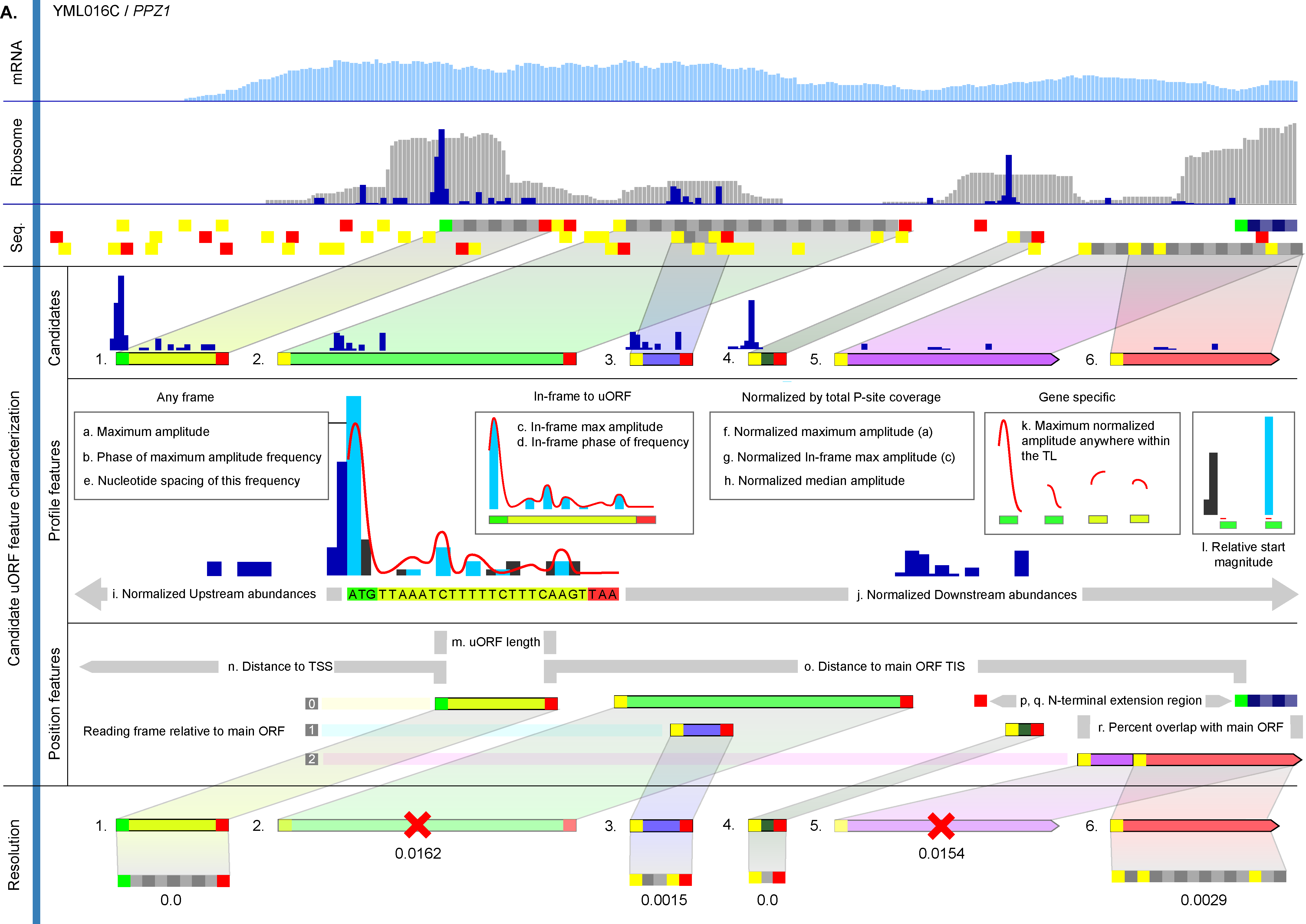


**Figure S4. Overview of uORF-scoring and resolution using uORF-seqr.** Representative figure of candidate uORF scoring using select features. Resolution of nested (uORFs 2,3) and overlapping (uORFs 5,6) uORFs relies on features and final q-value resolution. See Supplemental Methods uORF-seqr regression feature definition).

**
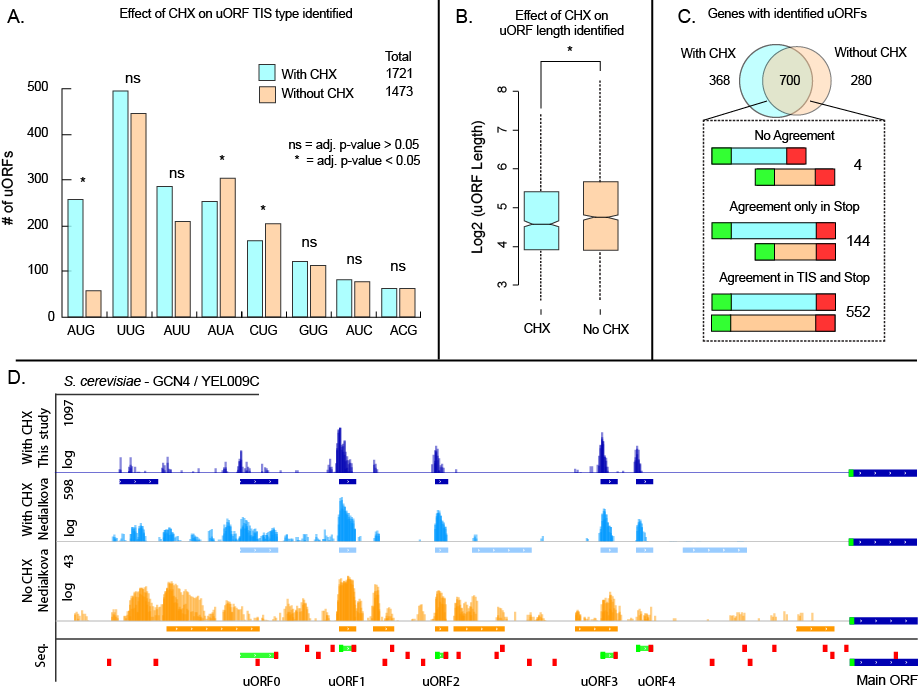
**

**Figure S5. CHX significantly improves detection of known uORFs using uORF-seqr.** (A) Using the Nedialkova dataset (Nedialkova 2015) we identify 248 more uORFs in the CHX treated samples than those without CHX, the majority of these (203) were AUG uORFs. (B) The median length of uORFs predicted from the no-CHX sample were 1.125 fold longer. (C) Comparison of uORFs predicted with and without CHX treatment. 202 of the 552 cases where identical uORFs were predicted with and without CHX initiated with UUG. The majority of uORF calls that disagreed on start codon location, shared the same reading frame and stop codon. This was most common for AUG initiating uORFs. (D) An example of the difference between the two treatments can be observed for the well characterized case of *GCN4*, which has 4 molecularly validated AUG uORFs and one NCC-type (Zhang 2011). All the known uORFs (green boxes) are called using CHX, while two are identified without CHX treatment and one predicts the wrong TIS site (yellow box). Two unverified uORFs are reported for the CHX treated sample (blue boxes), while four are reported for the untreated sample. Notably, despite similar read depths, the CHX untreated sample has much smaller peaks with more ribosomes located downstream of the TIS than observed with CHX. Thus, CHX treatment aids in robust identification of uORFs with high efficiency TISs, short lengths, or low ribosome coverage. Note that coverage in part D is on a log scale.


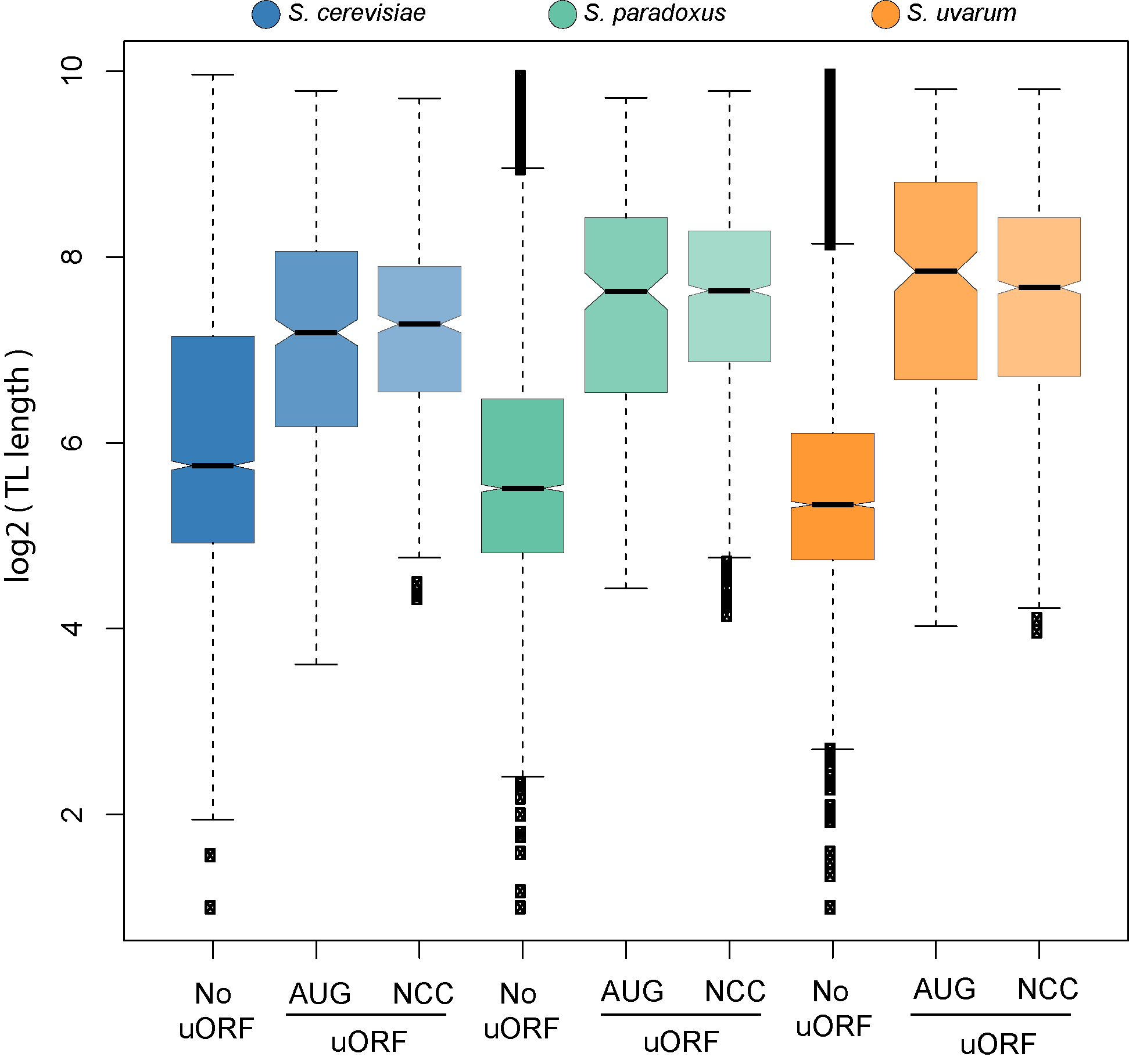

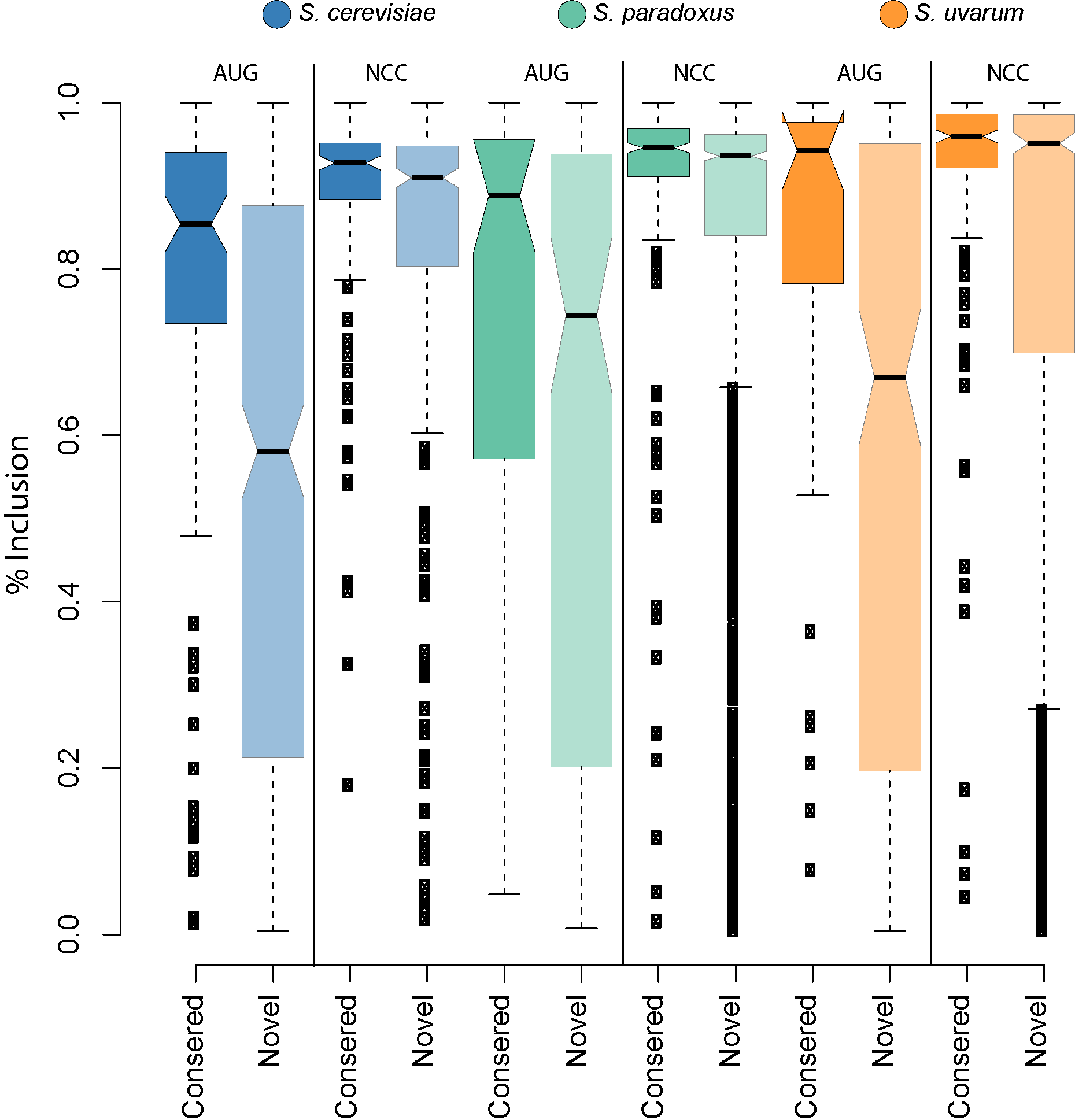


**Figure S6. Comparison of TIS and TL length.** (A) A comparison of the maximum length of TLs for genes with AUG uORFs, NCC uORFs, or no uORFs is shown. Although the distributions of TL lengths were the same for AUG and NCC uORFs, TL lengths for genes without uORFs were markedly shorter. (B) The rate of TIS inclusion for two types of AUG and NCC uORFs, those conserved in either sequence or position and those that were novel. Novel uORFs tend to have lower rates of inclusion especially for AUG uORFs (Wilcoxon Rank Sum Test, adj.p-value < 0.05). This suggests that AUG uORF innovation occurs primarily in alternative transcript leader regions.


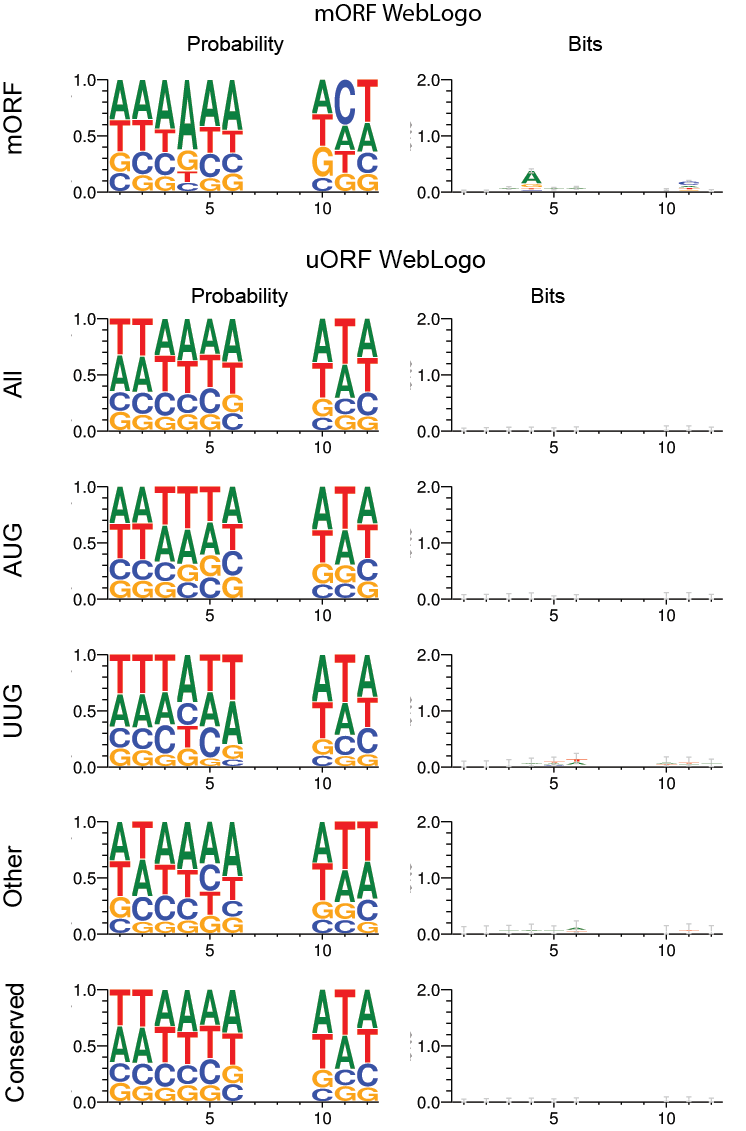


**Figure S7. Comparison of AUG and NCC uORF Kozak contexts.** The Kozak contexts for main ORFs, AUG-, and NCC-uORFs are plotted, showing nucleotide frequency (left, “Probability”) and information content (right, “Bits”) after correction for the AT-richness of the yeast genome. The yeast Kozak context is relatively weak for mORFs, and essentially absent for uORF start codons. Kozak contexts were plotted using weblogo (Crooks et al., 2004).


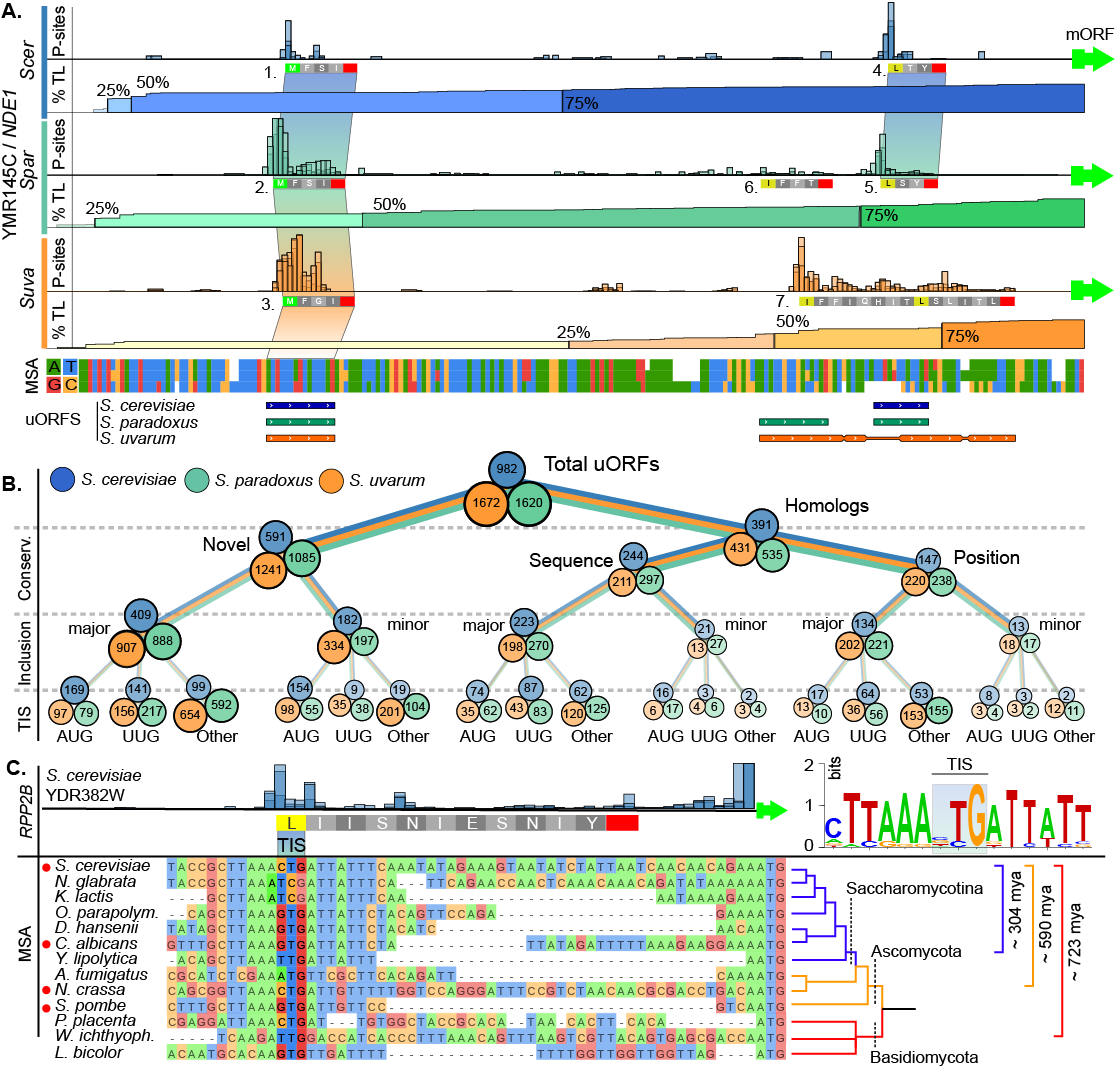


**Figure S8. Classification of sequence- and position-conserved uORFs.** (A) NDE1 is shown as an example gene containing uORFs conserved by sequence or position. Vertical peaks represent P site abundance per nucleotide, the horizontal gradient depicts the transcript leader percent inclusion. uORFs 1, 2, and 3 (left) are sequence homologs in all three species. Conversely, uORFs 4, 5, and 6 are not homologous with uORF 7 (right) as they lack significant overlap with uORF 7. For both S. cerevisiae and S. paradoxus, uORFs 1 and 2 are incorporated in the majority of transcripts (>50%) while the homologous uORF in S. uvarum (3) is included in less than 25% of the transcripts. (B) Flow-chart classifying uORFs based on their conservation (novel or homologous), whether or not they are included in the majority of transcripts (major uORFs) or in a minority (minor uORFs), and their start codon (AUG, UUG, or Other). Major uORFs occur have similar frequency of AUG and UUG start codons, while minor uORFs are more likely to start with AUG. (C) An NCC uORF in RPP2B is deeply conserved in fungi. The conservation of the NCC TIS of RPP2B across thirteen species of fungi, representing ~700 my divergence (Hedges et al. 2015) is shown. Species marked with a red circle have the proposed TIS occupied by ribosomes in public ribo-seq data (See Figure S9). Note that the alignment depiction is trimmed to fit the figure. Original sequences and their alignment are provided in Supplemental Files 3A-D.


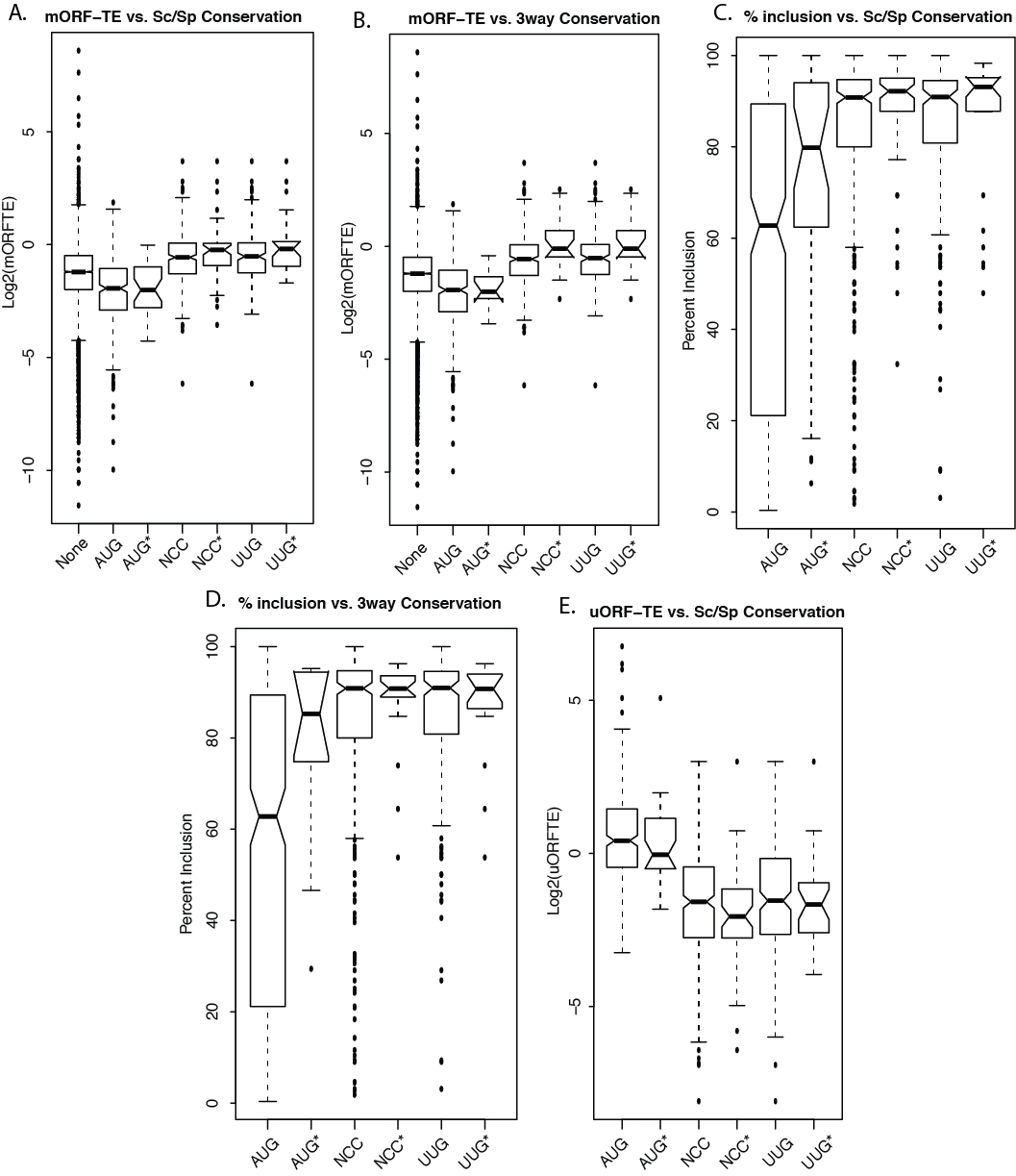


**Figure S9. Analysis of mORF-TE for sequence conserved AUG, UUG, and NCC uORFs.** Sequence conserved uORFs are indicated with asterices. (A) uORFs conserved between S. cerevisiae and S. paradoxus. NCC uORFs are associated with higher translation efficiency. (B) A similar results is observed for uORFs conserved between all three species. (C) Sequence conserved NCC uORFs (S. cerevisiae vs. S. paradoxus) have higher percent inclusion than AUG uORFs. (D) A similar result is seen for uORFs conserved between three species (including S. uvarum). (E) NCC uORFs (conserved between S. cerevisiae and S. paradoxus) have lower translation efficiency than AUG uORFs.

­­­­­
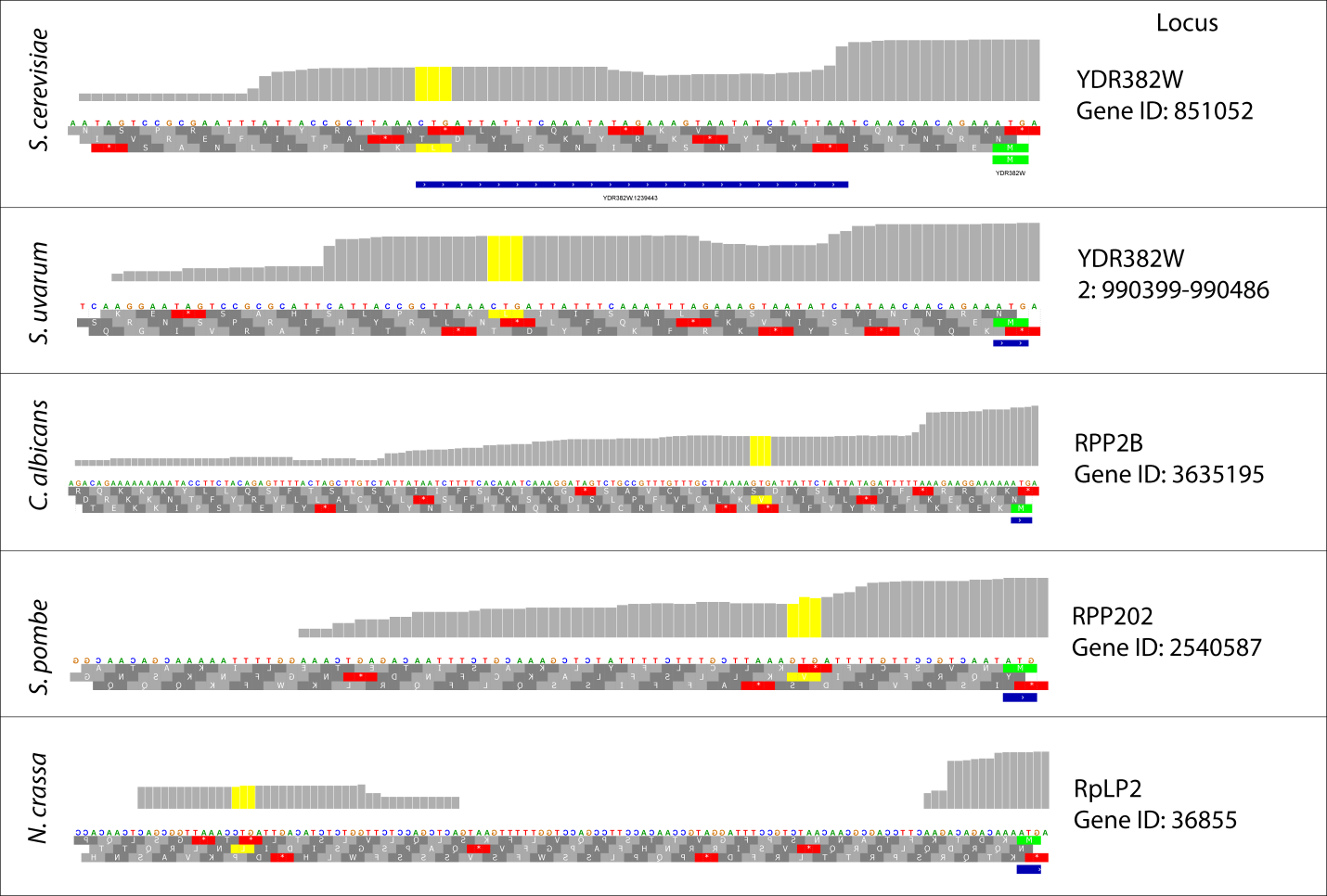


### Figure S10. Ribosome occupancy upstream of RPP2B homologs in other fungal species. Publicly available (NCBI GEO) ribosome profiling data are shown (*Candida albicans*: GSE52236, *Schizosaccharomyces pombe*: E-MTAB-2179, *Neurospora crassa*: GSE71032) alongside our data for *S. cerevisiae* and *S. uvarum*. In each case ribosomes are found over the aligned proposed TIS.


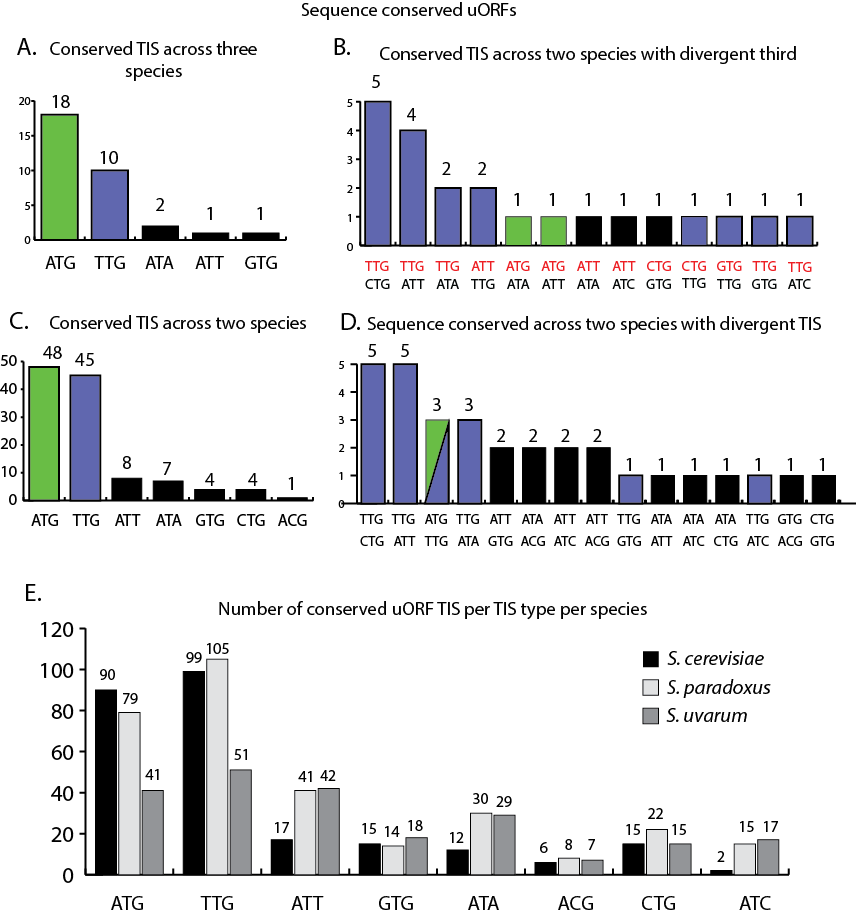


**Figure S11. uORF conservation by TIS type.** The above bar graphs indicate numbers of sequence conserved uORFs initiating with various start codons. (A) uORFs that have the same start codon in all three species. (B) uORFs that have the same start codon (red, upper) in two species, with the indicated third having the start codon indicated below. (C) uORFs that have the same start codon in two species. (D) uORFs that show conserved sequence (see methods) in two species, but have different start codons. (E) Number of sequence conserved uORFs with each type of start codon in each species.

**
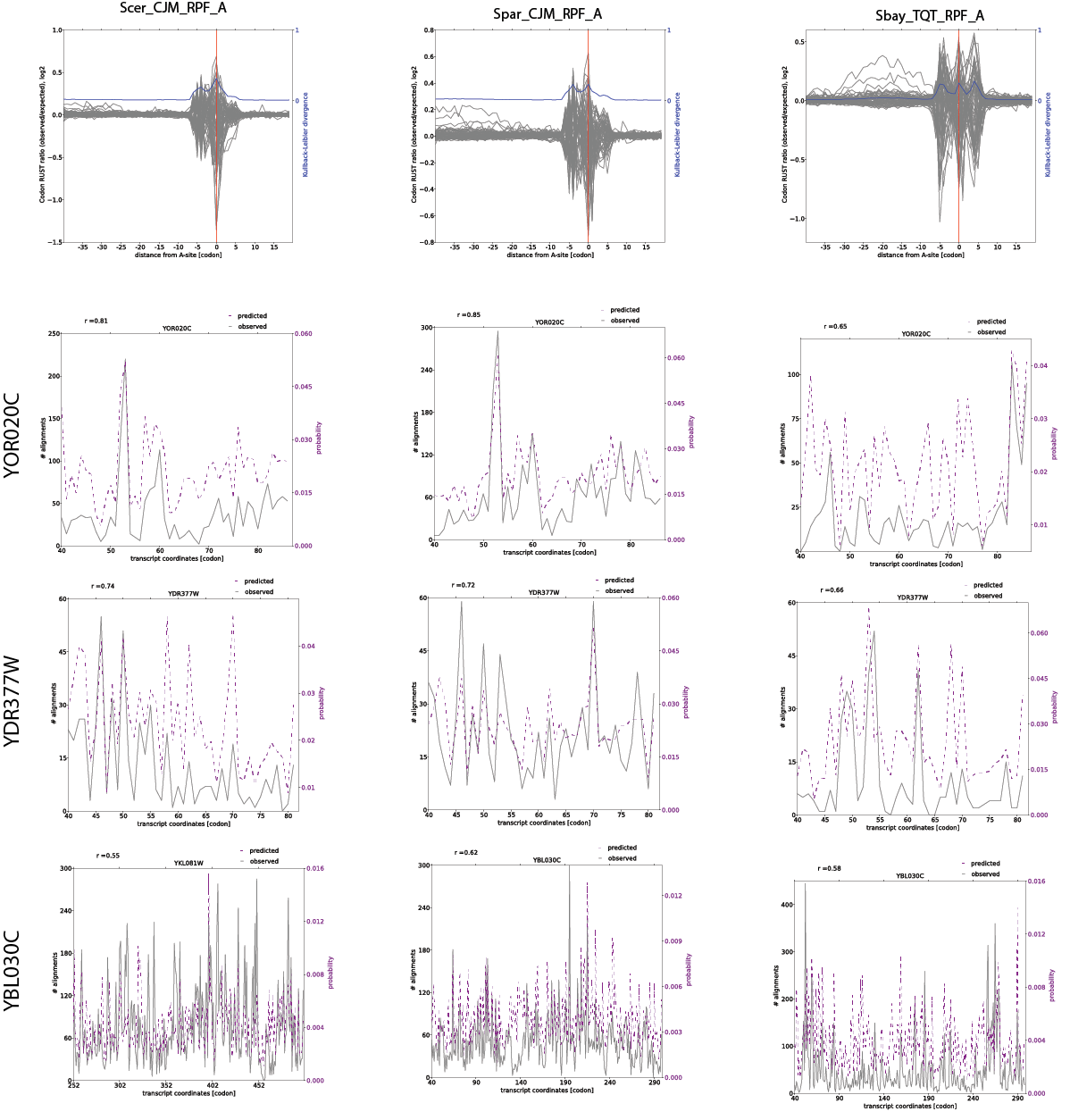
**

**Figure S12. RUST analysis comparison of Ribo-seq biases among datasets**. The top row plots show the results of RUST analysis for the first replicate of each dataset (*S. cerevisiae* left; *S. paradoxus* center*, S. uvarum* right). Lower rows show example comparisons of the expected and actual ribo-seq coverage seen across several genes.


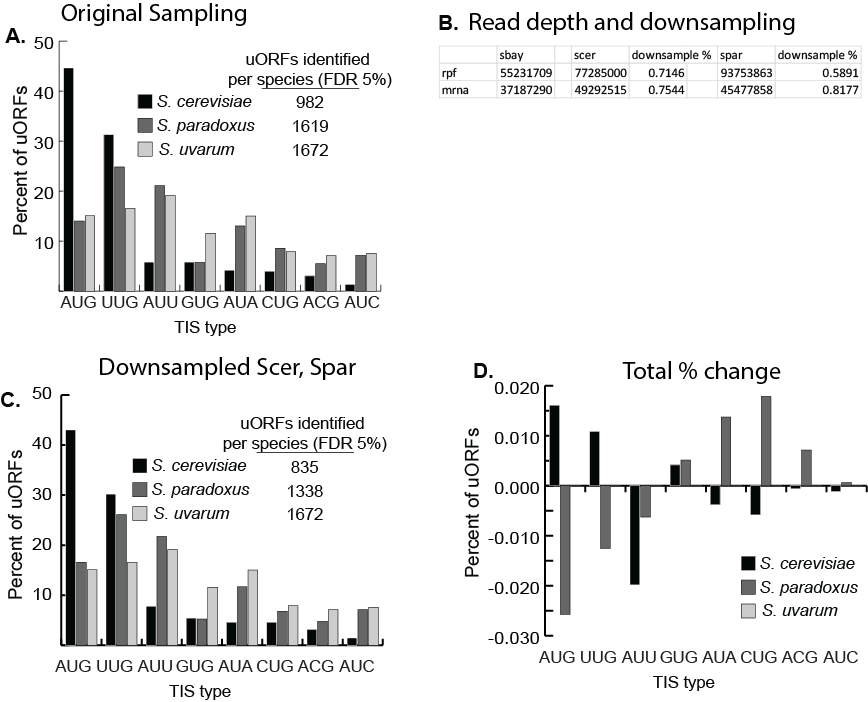


**Figure S13. Impact of sequencing depth on uORF-seqr predictions**. The number of RNA-seq and Ribo-seq reads differed between species. We investigated how this might affect uORF-seqr predictions, and found minimal change when downsampling reads from *S. cerevisiae* and *S. paradoxus* to match the depth obtained for *S. uvarum*. (A) Number of uORFs identified with each start codon type using the original datasets. (B) Table showing the total rpf and mRNA reads for *S. uvarum* (sbay), *S. cerevisiae* (scer) and *S. paradoxus* (spar). The downsampling percentage indicates the amount of downsampling performed on *S. cerevisiae* and *S. paradoxus* datasets to match the depth of *S. uvarum*. (C) uORF-seqr results after down-sampling *S. cerevisiae* and *S. paradoxus* datasets. Although fewer uORFs were called after down-sampling, the relative numbers of AUG and NCC uORFs changed very little. (D) The change in the percent of uORF calls for each start codon type is shown.

**
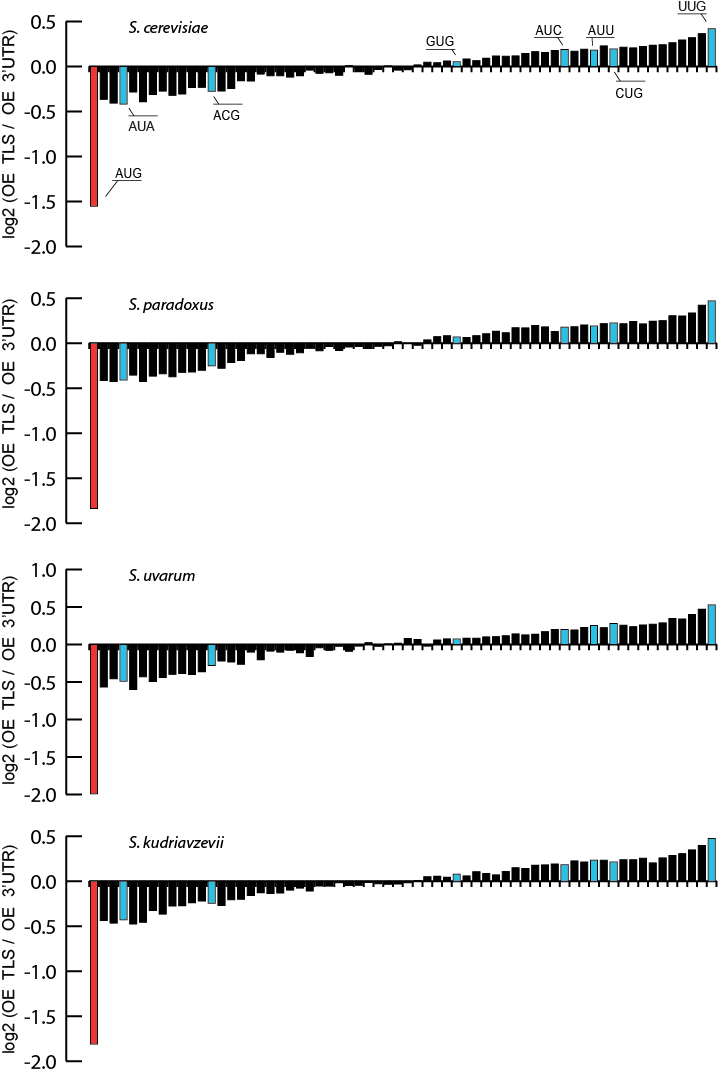
**

**Figure S14. Triplet frequency rates in transcript leaders from *Saccharomyces* yeast.** The plots above show the observed / expected rates of triplet sequences for AUG and NCC start codons in 5’ transcript leaders from each of the four species included in this study. Triplets are plotted in the order of enrichment shown in Figure 2C.

**Supplemental Tables:**

**Table S1. Gene Ontology term enrichment in conserved short and long transcript leaders.** GO term enrichment was assessed using *Saccharomyces* Genome Database’s Yeastmine. Transcript leader length conservation was assessed by calculating the coefficient of variation (CV) of leader lengths across *S. cerevisiae*, *S. paradoxus*, *S. kudriavzevii*, and *S. uvarum*. Genes with the lowest decile of CV values were considered as having particularly well conserved leader lengths. Of these, short (<=55 nt) leaders were enriched in functions involving translation (GO FDR <0.05) and glucose fermentation (KEGG and reactome FDR < 0.05), while those with long (>55 nt) TLs were enriched in numerous cell signaling GO categories (all at BH-corrected FDR = 0.05). For full results, see file Supplemental_table_S1.xlsx.

| MSA Count | ATG | GTA, GAT, TAG, TGA, AGT |
| --- | --- | --- |
| Conserved | 29,294 | 100,462 |
| Diverged | 53,772 | 226,927 |

### Relative Conservation Ratio: ATG is 1.23-fold more conserved, adj.p-value <0.05

**Table S2. Conservation rates of ATG in TLS.** The rate of ATG triplets conserved in local alignments between the TLs of *S. cerevisiae*, *S. paradoxus*, and *S. uvarum* is 1.23-fold higher than the conservation rate of the permutation of the same three nucleotides (GTA, GAT, TAG, TGA, and AGT). TLs used were the maximum TL lengths reported in the genome annotations of each species (Supplementary Files 1A-D). Local alignments were performed using BLASTn (ver. 2.2.25, Zhang et al 2000) with a 1/-5 penalty. Conserved alignments required the entire triplet be conserved without gaps. Equivalent counts were performed for all sets of triplets (Supplemental Files 4)

| Data Set | Total uORFs | Genes w/ uORFs | Overlap  By Gene | Nucleotide  Match | Near-Nucleotide Match | Median Length |
| --- | --- | --- | --- | --- | --- | --- |
| Cvijović | 2151 | 257 | 64 | 38 | 41 | 36 |
| Selpi | 398 | 301 | 77 | 44 | 46 | 18 |
| Lawless | 1481 | 554 | 154 | 105 | 129 | 36 |
| Ingolia | 296 | 243 | 150 | 78 | 89 | 50 |
| Brar | 10,159 | 3,121 | 698 | 416 | 494 | 32 |

**Table S3. Comparison of uORF-seqr and other published uORF predictions.** Previous work to identify *S.* cerevisiae uORFs has either relied on purely computational approaches (Cvijović et al. 2007, Selpi et al. 2009, Lawless et al. 2009) or heuristic analysis of ribosome profiling data (Ingolia et al. 2009, Brar et al. 2011). Our predictions overlap with previous published predictions to varying degrees. We predicted uORFs for roughly 25% (median) of the same genes previously predicted. This drops to a median of 7% agreement for uORFs with an exact nucleotide match. This increases to 9% if near-nucleotide (+/-3nt) matches are also included. The agreement is highest between our set and that of Ingolia et al, with an exact match of 26% of all predictions.

### Table S4. uORF-seqr uORF predictions with TIS type and Homology type. Each uORF, from each species, is listed along with the TIS type (AUG, NCC, UUG, Other) and homolog type (Sequence Conserved, Position Conserved Start and Stop, and Minor or Major TSS uORF), (see Supplemental_Table_S4.xlsx).

| Descriptor | Benjamani-Hochberg adj. p-value | Number of Matches |
| --- | --- | --- |
| fungal-type cell wall organization or biogenesis [GO:0071852] | 2.94E-04 | 47 |
| fungal-type cell wall organization [GO:0031505] | 8.46E-04 | 38 |
| cell wall organization or biogenesis [GO:0071554] | 0.001098 | 53 |
| external encapsulating structure organization [GO:0045229] | 0.001245 | 43 |
| cell wall organization [GO:0071555] | 0.001245 | 43 |

**Table S5. Enrichment of GO terms in genes with sequence or positional homolog uORFs compared to all genes with uORFs**. GO term enrichment was performed using *Saccharomyces* Genome Database’s Yeastmine (accessed January 11, 2017). GO enrichment was assessed for genes with at least one uORF with either sequence or positional homology (438). The background gene list includes genes from any species with at least one uORF (1549). All GO term enrichments with adj. p-values of less than 0.05 are shown.

**Table S6**. Regression weights for uORF-seqr models from different datasets. We note that, although weighted highly in all three regressions, the relative start magnitude feature is correlated with the other features in complex ways. As a result, this feature alone often failed to identify known molecularly validated uORFs.

| **Feature** | **Description** | ***S. paradoxus*** | ***S. uvarum*** | ***S. cerevisiae*** |
| --- | --- | --- | --- | --- |
| NA | *Intercept* | 0.32427 | 0.72398 | 0.33340 |
| **a** | *within_power_of_max_power_freq* | -0.06810 | 0.00000 | 0.00000 |
| **b** | *abs_phase_of_max_power_freq* | -0.07538 | -0.06953 | 0.12659 |
| **c** | *within_power_of_in_frame* | -0.14203 | -0.10346 | -0.09108 |
| **d** | *abs_phase_of_in_frame* | -0.05534 | -0.08724 | 0.05367 |
| **e** | *spacing_of_max_power* | -0.00760 | 0.00000 | -0.01306 |
| **f** | *relative_power_of_max_power_freq* | 0.03594 | 0.00010 | 0.00000 |
| **g** | *relative_power_of_in_frame* | 0.09826 | 0.03138 | 0.14349 |
| **h** | *relative_median_power* | 0.03560 | 0.00000 | -0.22706 |
| **i** | *relative_avg_upstream_reads* | -0.04061 | -0.39496 | -0.02504 |
| **j** | *relative_avg_downstream_reads* | -0.29334 | 0.01602 | -0.09595 |
| **k** | *region_relative_morf_peak_power* | -0.33366 | -0.19562 | -0.04810 |
| **l** | *normalized_start_magnitude* | 0.62308 | 0.54765 | 4.64378 |
| **m** | *length_of_puorf* | -0.15625 | -0.04148 | -0.02959 |
| **n** | *morf_dist_TIS* | -0.00174 | -0.03576 | 0.05246 |
| **o** | *morf_dist_TSS* | 0.00973 | -0.05746 | 0.05383 |
| **p** | *morf_nterm_region_start_after* | 0.05614 | 0.03669 | 0.05270 |
| **q** | *morf_nterm_region_ends_before* | -0.04787 | -0.02321 | -0.03370 |
| **r** | *morf_nonoverlap* | -0.07924 | 0.00000 | -0.03086 |

**Table S7**. List of sequence and position conserved uORFs. See file “Supplemental_Table_S7.xlsx”.Works cited:

Brar GA. et al. 2011. High-Resolution View of the Yeast Meiotic Program Revealed by Ribosome Profiling. Science 335: 552–557.

Chu D., Kazana E., Bellanger N., Singh T., Tuite M. F., Haar von der T. 2013. Translation elongation can control translation initiation on eukaryotic mRNAs. *EMBO J*, *33*(1), 21–34.

Crooks GE. et al. 2004. “WebLogo: A sequence logo generator”, *Genome Research*, 14:1188-1190

Cvijović M. et al. 2007. Identification of putative regulatory upstream ORFs in the yeast genome using heuristics and evolutionary conservation. BMC Bioinformatics 8: 295.

Dobin A, Davis CA, Schlesinger F, Drenkow J, Zaleski C, Jha S, Batut P, Chaisson M, Gingeras TR. 2013. STAR: ultrafast universal RNA-seq aligner. *Bioinformatics* **29**: 15–21.

Edgar RC. 2004. MUSCLE: multiple sequence alignment with high accuracy and high throughput. Nucleic Acids Res. 32(5):1792

Ingolia NT, et al. 2009. Genome-wide analysis in vivo of translation with nucleotide resolution using ribosome profiling. Science 324: 218–223.

Lawless C. et al. 2009. Upstream sequence elements direct post-transcriptional regulation of gene expression under stress conditions in yeast. BMC Genomics 10:7.

McManus CJ, May GE, Spealman P, Shteyman A. 2014. Ribosome profiling reveals post-transcriptional buffering of divergent gene expression in yeast. *Genome Res* **24**: 422–430.

Nedialkova DD, Leidel SA. 2015. Optimization of Codon Translation Rates via tRNA Modifications Maintains Proteome Integrity. Cell 161: 1606–1618.

Ramdas A, Tibshirani R. 2016. Fast and Flexible ADMM Algorithms for Trend Filtering.*Journal of Computational and Graphical Statistics* **25**: 839-858.

Selpi, et al. 2009. Predicting functional upstream open reading frames in *Saccharomyces cerevisiae*. BMC Bioinformatics 10: 451.

Spealman P, Wang H, May G, Kingsford C, McManus CJ. 2016. Exploring Ribosome Positioning on Translating Transcripts with Ribosome Profiling. *Methods Mol Biol* **1358**: 71–97.

Strope, P. K. et al. The 100-genomes strains, an S. cerevisiae resource that illuminates its natural phenotypic and genotypic variation and emergence as an opportunistic pathogen. Genome Res. 25, 762–74 (2015).

Tibshirani et al., 2001 Estimating the number of clusters in a data set via the gap statistic. J. R. Statist. Soc. B. 63: 411-423.

Waterhouse AM. et al. 2009 Jalview Version 2-a multiple sequence alignment editor and analysis workbench. Bioinformatics 25: 1189-1191.

Zhang Z. et al. 2000. A greedy algorithm for aligning DNA sequences. J Comput Biol. Feb-Apr; 7(1-2).

Zhang F., Hinnebusch AG. 2011. An upstream ORF with non-AUG start codon is translated in vivo but dispensable for translational control of GCN4 mRNA. Nucleic Acids Research: Apr;39(8):3128-40.

Arribere JA, Gilbert WV. 2013. Roles for transcript leaders in translation and mRNA decay revealed by transcript leader sequencing. *Genome Res* **23**: 977–987.
